# Characterisation of aptamer–target interactions by branched selection and high-throughput sequencing of SELEX pools

**DOI:** 10.1093/nar/gkv700

**Published:** 2015-07-10

**Authors:** Daniel M. Dupont, Niels Larsen, Jan. K. Jensen, Peter A. Andreasen, Jørgen Kjems

**Affiliations:** 1 Department of Molecular Biology and Genetics, Aarhus University, 8000 Aarhus C, Denmark; 2 Danish-Chinese Centre for Proteases and Cancer, Aarhus University, 8000 Aarhus C, Denmark; 3 iNANO Interdisciplinary Nanoscience Center, Aarhus University, 8000 Aarhus C, Denmark

## Abstract

Nucleic acid aptamer selection by systematic evolution of ligands by exponential enrichment (SELEX) has shown great promise for use in the development of research tools, therapeutics and diagnostics. Typically, aptamers are identified from libraries containing up to 10^16^ different RNA or DNA sequences by 5–10 rounds of affinity selection towards a target of interest. Such library screenings can result in complex pools of many target-binding aptamers. New high-throughput sequencing techniques may potentially revolutionise aptamer selection by allowing quantitative assessment of the dynamic changes in the pool composition during the SELEX process and by facilitating large-scale post-SELEX characterisation. In the present study, we demonstrate how high-throughput sequencing of SELEX pools, before and after a single round of branched selection for binding to different target variants, can provide detailed information about aptamer binding sites, preferences for specific target conformations, and functional effects of the aptamers. The procedure was applied on a diverse pool of 2′-fluoropyrimidine-modified RNA enriched for aptamers specific for the serpin plasminogen activator inhibitor-1 (PAI-1) through five rounds of standard selection. The results demonstrate that it is possible to perform large-scale detailed characterisation of aptamer sequences directly in the complex pools obtained from library selection methods, thus without the need to produce individual aptamers.

## INTRODUCTION

Library screening methodologies such as phage display and systematic evolution of ligands by exponential enrichment (SELEX) are useful tools for the identification of novel protein-targeting ligands for pharmacological intervention, drug delivery, molecular imaging, and other diagnostic and prognostic analysis (1–4). In SELEX experiments, up to 10^16^ unique nucleic acid sequences are screened for their ability to bind a target of interest. The general frequency of target binding nucleic acid molecules (aptamers) in random libraries is in the order of 10^−10^–10^−14^ for most proteins (5,6), and through iterative rounds of affinity selection and amplification of the pool, the relative content of target-binding aptamers is increased to a level where they can be identified by sequencing a limited number of clones. The strategy has in many cases allowed the identification of aptamers with high affinity (pM - nM range) and high specificity for their protein targets (3,7).

Post-SELEX characterisation of aptamers is usually the most laborious task, limiting the number of candidates subjected to detailed investigations. To decrease the number of candidates the SELEX process traditionally is repeated many times until only few candidates are dominating the pool. However, SELEX pools earlier in the selection may contain thousands of useful aptamers, many of which may remain undetected, either because they become extinct during the selection procedure or because the complexity of their ancestors is so high that the enrichment becomes delayed. Moreover, even though selections can be directed to different sites of the target protein by varying the selection conditions, it is rarely done. Novel strategies are therefore needed to facilitate the identification of aptamers from rare sequence families with desirable binding properties and functional effects.

High-throughput sequencing (HTS) technologies have revolutionised data collection in SELEX experiments enabling quantitative insights into the dynamic process of sequence enrichment during aptamer selection experiments (8–10). In contrast to the general perception, the most abundant sequences of the final round of a selection experiment are not necessarily the ones with highest target affinity (8,11). In fact, determining round-to-round enrichment of a particular aptamer sequence in early rounds of selection can result in the identification of higher affinity aptamers, which, as another trade-off, reduces the number of selection rounds needed, PCR bias, and artefact selection (8,11). Furthermore, large sequence data sets enable a more robust structure prediction from covariance analysis and comparison of sequence variations with target affinities (8,11–13). Such information is useful for guiding aptamer truncation without loss of target affinity (10).

We reasoned that in addition to being able to determine the relative binding affinities of all aptamers in a SELEX pool, HTS-based data analyses may also provide information about aptamer binding sites and functional effects. We here present a procedure for mapping protein binding sites of multiple aptamers in enriched SELEX pools as well as determining their competition with natural protein target ligands. The protocol was applied on a previous selection experiment for 2′-fluoropyrimidine-modified (2′-F-Y) RNA aptamers binding to the serpin plasminogen activator inhibitor 1 (PAI-1) (14). PAI-1 is involved in fibrinolysis as well as in normal and pathological tissue-remodelling events, through its role as the primary physiological inhibitor of the two serine proteases, tissue-type plasminogen activator (tPA) and urokinase-type plasminogen activator (uPA), respectively (15). In our previous selection experiment for PAI-1 aptamers, two dominating aptamers (paionap-5 and -40) in the final pool were characterised in biochemical detail (14,16). Using a combination of site-directed mutagenesis and functional assays, the binding sites of the two aptamers were mapped and functional effects determined. PAI-1 is a metastable protein that spontaneously converts into a non-inhibitory, so-called latent form, by a large intramolecular conformational change. In spite of overlapping binding sites, only paionap-5, but not paionap-40, was able to bind both conformations with measurable affinity. In agreement with the identified binding sites, the two aptamers inhibited the interaction of PAI-1 with the extracellular matrix protein vitronectin, the presumed *in vivo* binding partner of PAI-1 in the blood and tissue compartments.

In the present study, we demonstrate that HTS of SELEX pools, before and after a parallel binding selection to a panel of alanine mutants of PAI-1, enables individual and global characterisation of binding sites for the aptamers in a pool enriched for target-binding RNA. Furthermore, applying distinct conformational forms of PAI-1 immediately reveal specificity of aptamers for target conformations. We also demonstrate that a single round of parallel selection towards the complex formed between PAI-1 and vitronectin enables prediction of aptamers inhibiting this interaction. Interestingly, although the vitronectin binding site of PAI-1 is a hotspot for aptamer binding, the procedure readily allows the identification of underrepresented aptamers in the pool, binding outside this area. Finally, multiple alignments of aptamer family members enabled the determination of aptamer structure-function features corresponding well with biochemical data. The approach outlined here facilitates rapid characterisation and sorting of aptamers from SELEX pools with a minimum of labour.

## METHODS AND MATERIALS

### Proteins and RNA library construction

For amino acid residues in PAI-1, we will refer to the numbering system of Andreasen *et al*. (17) starting with Ser^1^-Ala^2^-Val^3^-His^4^-His^5^. Glycosylated natural human PAI-1 was purified from serum-free conditioned medium of dexamethasone-treated HT-1080 cells by immunoaffinity chromatography (14). Purified PAI-1 was in the latent form and active preparations prepared by denaturation with guanidinium hydrochloride and refolding by dialysis against phosphate-buffered saline (PBS; 10 mM NaH_2_PO_4_ pH 6.9, 140 mM NaCl). Preparations of active PAI-1 (90–95% pure) were obtained by affinity chromatography using immobilised β-anhydrotrypsin. Recombinant PAI-1 variants (wild type and mutants with single alanine substitutions) with an N-terminal hexa-His tag were expressed in *Escherichia coli* (*E. coli*) at 30°C to minimise latency transition, and purified by nickel affinity chromatography followed by size exclusion chromatography on a Superdex 75 column (14). The PAI-1 mutants were produced by site-directed mutagenesis using the QuickChange site-directed mutagenesis kit (Stratagene). All the tested PAI-1 mutants folded into the active conformation as verified by specific inhibitory activities (18) of around 50–80% of the theoretical maximum. PAI-1 in the active or the latent conformations was confirmed by the ability or inability to form a complex with a molar excess of uPA (Wakamoto) as analysed by SDS-PAGE.

The DNA transcription template for producing the 2′-F-Y RNA SELEX pool partially enriched for PAI-1 binding aptamers was generated by PCR-amplification (Thermo Scientific) of the original SELEX template obtained after five rounds of affinity selection with PAI-1 as the target (14). Templates for individual RNA sequence production were produced by Klenow extension (Klenow Fragment exo-system; Thermo Scientific) of the standard primer sets used for library preparation as described (14), but with specified random regions. Templates were purified by 6% non-denaturing gel electrophoresis (National Diagnostics) and 2′-F-Y RNA produced in reactions containing 80 mM HEPES (pH 7.5), 30 mM DTT, 25 mM MgCl_2_, 2 mM spermidine-HCl, 2.5 mM ATP and GTP (Thermo Scientific), and 2.5 mM 2′-F-dCTP and 2′-F-dUTP (TriLink Biotechnologies), 100 μg/ml BSA (Thermo Scientific), 0.5–1 μM dsDNA template, and 150 μg/ml mutant T7 RNA polymerase Y639F. RNA transcripts were purified by 8% denaturing polyacrylamide gel electrophoresis (National Diagnostics) as described earlier (14). The uPA aptamer upanap-12 (19) was used as control RNA.

### Microtiter plate parallel selection with the input RNA pool

Wells of 96-well microtiter Maxisorp plates (NUNC) were coated overnight with a rabbit polyclonal anti-PAI-1 antibody (5 μg/ml), the monoclonal anti-PAI-1 antibody mAb-1 (5 μg/ml) binding in α-helix C (20), or monomeric vitronectin (1 μg/ml; Molecular Innovations) using 0.1 M Na_2_CO_3_/NaHCO_3_ pH 9.6. Wells were blocked with 1% bovine serum albumin (BSA; Sigma–Aldrich) in HEPES-buffered saline (HBS; 10 mM HEPES pH 7.4, 140 mM NaCl). PAI-1 variants (20 μg/ml) in HBS with 0.1% BSA and 0.01% Tween20 were captured onto antibody or vitronectin for 1 h at room temperature followed by washing. For the analysis of aptamer binding sites different single substituted alanine PAI-1 variants were used. For estimation of conformational preferences and binding to the PAI-1:vitronectin complex, glycosylated PAI-1 purified from HT-1080 cells was used. Aliquots of the RNA pool (100 nM) in binding buffer (HBS, 2 mM MgCl_2_, 2 mM CaCl_2_, 0.1% BSA) were first added to coated wells without PAI-1 and then transferred to coated wells with PAI-1. After 45 min of incubation, wells were washed six times with binding buffer. RNA was recovered by incubation with proteinase K (25 μg; Sigma–Aldrich) in 10 mM Tris–HCl, pH 7.8, 5 mM EDTA, 0.5% SDS for 30 min at 37°C, followed by phenol–chloroform extraction, and precipitation.

### High-throughput sequencing of samples

RNA was reverse transcribed using the reverse primer (5′-CCCGACACCCGCGGATCCATGGGCACTATTTATATCAA-3′) and RevertAid Premium reverse transcriptase (Thermo Scientific). dsDNA samples for sequencing were prepared by PCR (Native Pfu DNA polymerase; Thermo Scientific) and purified using GeneJet PCR purification kit (Thermo Scientific). For each PCR sample forward and reverse standard selection primers (14) containing specific barcode sequences were used. Barcoded PCR samples were mixed in equimolar amounts and submitted to the Beijing Genomics Institute (BGI) for PCR-free library preparation and one-lane 2×90 (Paired End) Illumina HiSeq 2000 sequencing.

### HTS data processing and analysis

Forward and reverse reads were sorted according to their specific barcoding (demultiplexing). Reads were included that contained correct constant regions, a variable region between 25 and 45 nucleotides and for which a forward-reverse pair was found. Pairs were combined and constant regions removed leaving only the variable region sequences. Sequences were then replicated and clustered at 90% identity to obtain copy numbers. A table of the sequences, each with a specific ID code, along with copy numbers in each PCR sample was generated and imported into Microsoft Excel for further analysis. Generally, a total of one to two million read pairs were obtained from each sample. Sequence copy numbers for each sample were divided by the total read number to estimate sequence frequency as the fraction of pool in percent allowing comparison of values across samples. The MEME suite was used for identifying conserved motifs among RNA sequences (21,22). Following settings were used: Number of sites (10–300), zero or one motif occurrence per sequence, motif width (5–40), maximum number of different motifs (25), search given strand only. Motif sequences extracted were based on >90% conservation for each nucleotide position. Secondary structure predictions for individual RNA sequences were performed with RNAshapes using standard settings (24,25). For LocARNA alignment we used standard settings (global, standard). LocARNA is a tool for multiple alignment of RNA molecules and simultaneously folds and aligns the input sequences (23).

### RNA binding to PAI-1 by surface plasmon resonance analysis (SPR)

Aptamer-PAI-1 binding was studied with a BIACORE T200 (GE Healthcare) using biotinylated aptamers captured on sensor surfaces coupled with 500 response units (RU) streptavidin (Sigma–Aldrich; 10 μg/ml in 10 mM NaOAc pH 4.5). Upanap-12 (19) was used as a negative control aptamer. Biotinylation of aptamers was performed by 3′-end ribose oxidation using sodium-metaperiodate followed by reaction with EZ-link Biotin-LC-Hydrazide using the standard protocol of the provider (Thermo Scientific). The sensor surfaces were regenerated after PAI-1 binding analysis (see results and figure text for details) with 50 mM EDTA in HBS. The running buffer was HBS supplemented with 1 mM MgCl_2_, 1 mM CaCl_2_, and 0.1% BSA. For studying binding of aptamers to the PAI-1:vitronectin complex CM5 sensor chip surfaces were also coupled with vitronectin (1000 RU; 30 μg/ml in 10 mM NaOAc pH 4). PAI-1 (10 nM) from HT-1080 cells was captured to a level of ∼100 RU on the vitronectin and the binding of 100 nM RNA to PAI-1 was recorded. Sensor surfaces were regenerated using 10 mM glycin–HCl pH 2.5 with 0.5 M NaCl.

## RESULTS

### Choice of input RNA pool

To investigate how selection for target variants can affect SELEX pool composition, we chose to work with an RNA pool from a previously reported experiment in which 2′-F-Y aptamers were selected against the PAI-1 protein (14). In the previous study, initial enrichment of PAI-1-binding RNA was detected following 5 rounds of selection, and dominating aptamers identified after 8 rounds of selection. In the present study, we focused on the highly diverse RNA pool after five rounds of selection (henceforth referred to as the input pool) and subjected it to different selection protocols in parallel (referred to as branched selection).

### Branched selection and HTS analysis procedure

**Figure 1.**
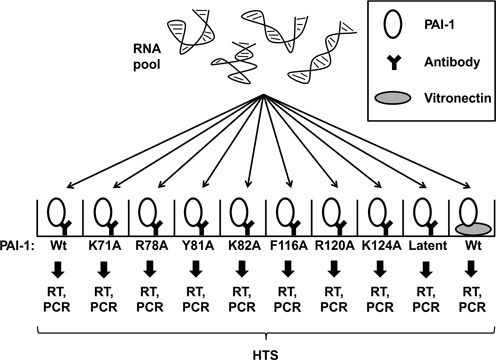
An overview of the method for parallelised aptamer characterisation. After five rounds of selection for binding to the serpin PAI-1, the enriched RNA pool was subjected to one round of parallel selections for binding to different PAI-1 variants (wild type and single-residue alanine mutants) and conformers (active and latent form) as well as PAI-1 presented by different antibodies or the natural extracellular matrix protein, vitronectin (VN). After a washing step, the retained RNA was reverse transcribed, PCR amplified with barcoded primers, and sequenced in one reaction by Illumina sequencing.

As outlined in Figure 1, a single round of selection was performed in parallel using either wild type or a variety of alanine substituted PAI-1 variants, all in the inhibitory active conformation. The PAI-1 variants were captured in a microtiter plate, by the use of either an immobilised monoclonal or polyclonal anti-PAI-1 antibody preparation. The pool was also screened for sequences binding to PAI-1 in the non-inhibitory latent conformation and for aptamers binding to inhibitory active PAI-1 captured on immobilised vitronectin. In each case, the RNA retained after washing was eluted, reverse transcribed, PCR amplified using primers with sample-specific barcodes and the products analysed by Illumina sequencing. We hypothesised that enrichment of specific input pool sequences would depend on target variant affinity and thereby provide information about aptamer binding sites, specificity for specific PAI-1 conformations, and effects on PAI-1 functions.

### Initial characterisation of the input RNA pool

**Figure 2.**
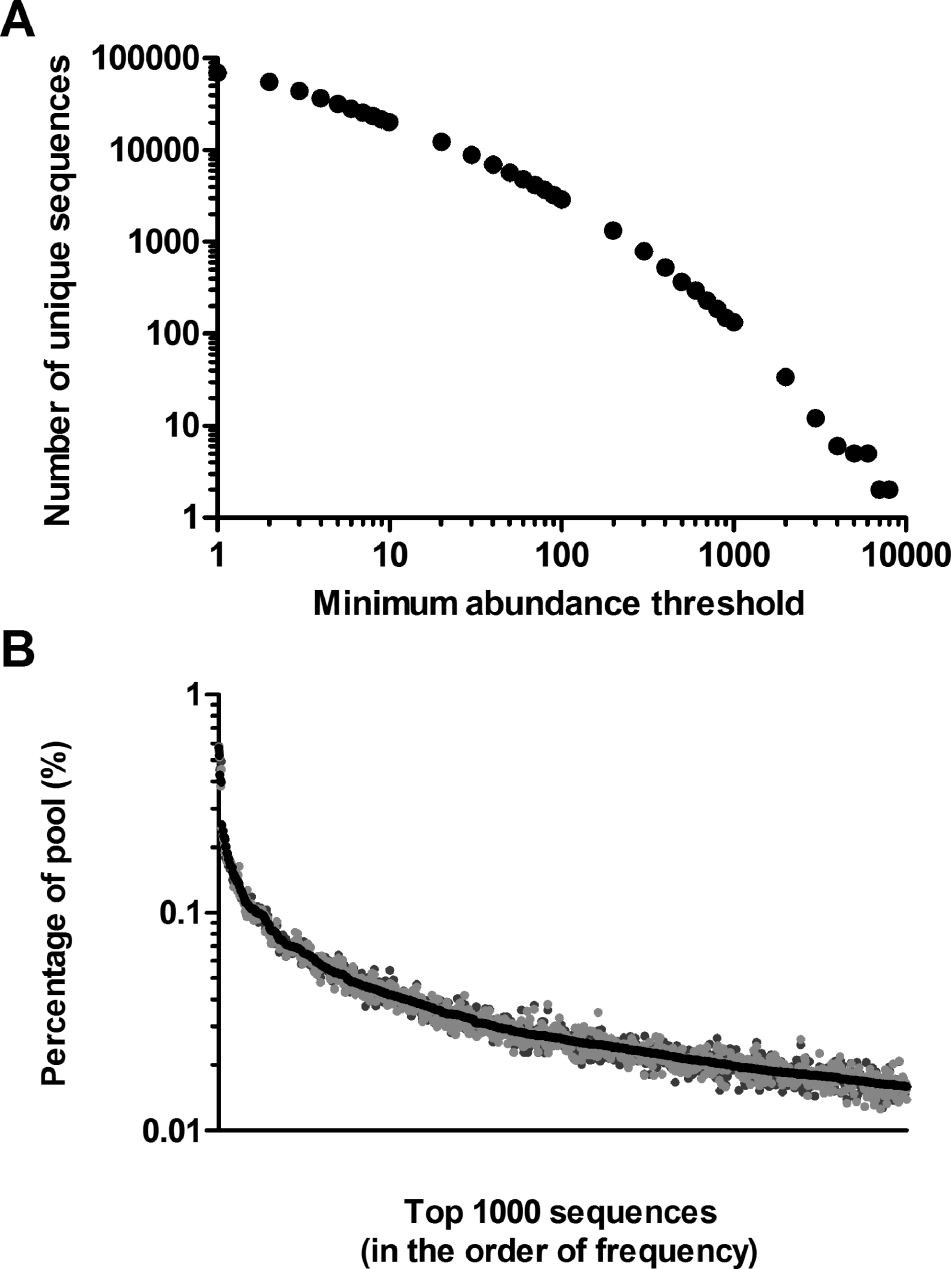
Characterisation of the input pool. (**A**) 69 945 unique sequences were observed at least one time. The graph depicts how the number of unique sequences decreases when increasing the minimum abundance threshold. For example, 20 206 sequences of the 69 945 were detected in the pool at least 10 times. (**B**) Sequence abundance as the percentage of pool (%) for the top 1000 most prevalent unique sequences in the input pool. Samples for HTS analysis were prepared three times in similar ways. Either from the re-amplified original SELEX template which was used for transcribing the RNA used in this study (input pool; black dots) or directly from the original SELEX template (Original #1; dark grey dots, and Original #2; light grey dots).

Sequencing of the input pool yielded a total of 1.58 × 10^6^ sequence reads representing 69 945 unique RNA sequences. Figure 2A illustrates how the number of unique sequences gradually decreases when increasing the minimum abundance threshold. The most recurring sequence (SEQ ID 2202) appeared 8 975 times and the 50 most abundant sequences of the input pool are shown in Supplementary Table S1. For each unique sequence, we calculated a ‘Percentage of pool’-value from the number of times it was observed in the pool relative to the total number of sequence reads. The most abundant unique sequence constitutes around half a percent of the input pool. Figure 2B graphically depicts the ‘Percentage of pool’-values for the top 1000 most frequently observed sequences in the input pool. The data illustrate the sequence diversity for the input pool, which was used for the branched selection procedure.

To obtain sufficient transcription template for RNA transcription, the original fifth round pool was re-amplified using 12 cycles of PCR. Comparing the sequencing result before (in replica, referred to as Original #1 and #2) and after this PCR amplification (the input pool), similar results for both the top 50 and top 1000 most frequently observed sequences were obtained (see ‘Percentage of pool’-values; Supplementary Table S1 and Figure 2B, respectively). The result shows that the PCR amplification did not lead to major differences in the relative composition of the pool. For the 50 sequences of Supplementary Table S1, the largest standard deviation based on the three samples was 11%, with the average being 4%, which demonstrates the experimental variability introduced by sample preparation and HTS analysis.

### Analysis of the changes in RNA pool composition after one round of branched selection against different target variants

**Figure 3.**
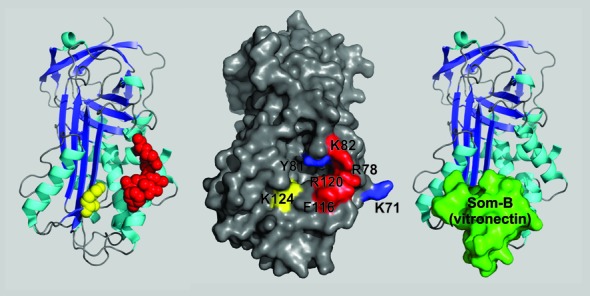
Binding sites of aptamers paionap-5 and -40 on PAI-1. Left panel: PAI-1 in a cartoon representation with residues important for binding of the aptamers shown as red (Arg78, Lys82, Phe116 and Arg120 recognised by both paionap-5 and -40) and yellow spheres (Lys124 recognised only by paionap-40) (14). Middle panel: Surface representation model of PAI-1 using same colour codes. Residues in blue (Lys71 and Tyr81) are unimportant for aptamer binding and were included in the branched selection-HTS analysis. Right panel: The position of the somatomedin B domain of vitronectin (green) when bound to PAI-1. The figure was prepared using PyMOL (the PyMOL Molecular Graphics System, Version 1.5.0.4 Schrödinger, LLC) and PDB ID: 1OC0.

We predict that the enrichment factor (EF_x_), defined as ‘Percentage of pool-value after selection to protein X’/‘percentage of pool-value before selection’, can be used as a measure of differential affinity to various PAI-1 mutants and hence provide information about the recognition site. To validate the method, we investigated the data for the two previously characterised PAI-1 aptamers paionap-5 and paionap -40 that have known binding sites on PAI-1 (Figure 3). In the previous study the binding site of both aptamers was found to include residues Arg78, Lys82, Phe116 and Arg120, while paionap-40 binding in addition recognises Lys124 (14).

Sequencing analysis of the input pool showed that paionap-5 was the fourth most abundant sequence (SEQ ID 551; Percentage of pool = 0.405%; Supplementary Table S1) and paionap-40 the 112th most abundant sequence (SEQ ID 53156; percentage of pool = 0.069%; data not shown). There was no correlation between sequence ranking and PAI-1:aptamer binding affinity as suggested by SPR analysis (data not shown). A single round of selection for binding to wild type PAI-1, immobilised on the microtiter plate using a polyclonal anti-PAI-1 antibody, increased the fraction of paionap-5 in the pool from 0.405% to 0.655%, corresponding to an EF_wt_ of 1.62 (Table 1 and Supplementary Table S1). In a similar way the EFs for paionap-5 with each PAI-1 variant were determined. To enable direct comparison, the EFs for each PAI-1 variant used were calculated relative to the wild-type case (EF_variant_/EF_wt_, Table 1). For PAI-1 mutations known to reduce the affinity of paionap-5 to PAI-1 (R78A, K82A, F116A, R120A) (14), the single round of selection resulted in a 5–6-fold reduction of the enrichment factor relative to wild type (Table 1). Similar results (7–10-fold reductions) were obtained when a monoclonal anti-PAI-1 antibody (mAb-1; Supplementary Table S2) was used for PAI-1 immobilisation. Therefore, for paionap-5, the previous reported reduction in binding affinity towards alanine mutants of PAI-1 correlates well with a reduction in enrichment factor.

**Table 1.** Sequences with a paionap-5 (SEQ ID 551) binding site

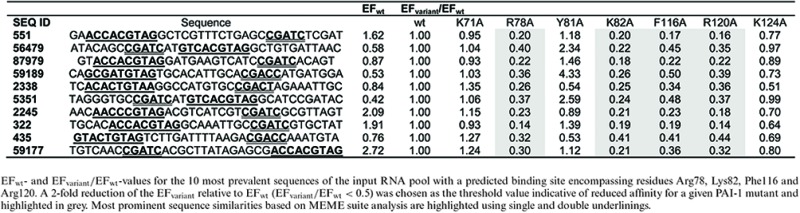										

Next, we scanned the input RNA pool sequences for unique sequences having a binding profile similar to that of paionap-5. In this operation, a more than two-fold reduction of EF_variant_/EF_wt_ was taken as a sign of reduced PAI-1 recognition for a particular mutant with a given sequence. We limited the scan to the 20 206 unique sequences observed at least 10 times in the input RNA pool (Figure 2A). Ninety-seven sequences were identified exhibiting a paionap-5-like recognition pattern, independently of whether a polyclonal or monoclonal anti-PAI-1 antibody was used. The EF_variant_/EF_wt_ data for the 10 most prevalent sequences in the input RNA pool (including paionap-5) out of the 97 sequences are shown in Table 1 and Supplementary Table S2, referring to each of the antibody immobilisation modes, respectively. We used the MEME suite (22) to search for sequence similarities among all 97 sequences. The analysis revealed two common consensus sequences, (A/G)(C/T)CACGTAG and CGATC, in 97 and 92 sequences, respectively (Supplementary Figure S1; motifs are also underlined in Table 1 and Supplementary Table S2). These motifs are also present in paionap-5. The results support the previous identification of the recognition site for paionap-5 and suggest that the two identified RNA sequence elements could be the PAI-1 recognition site in the RNA.

The same analysis was carried out for the paionap-40 aptamer (SEQ ID 53156). The results for paionap-40 after one round of selection with wild type PAI-1 and alanine mutants are shown in Table 2 (immobilisation on a monoclonal antibody) and Supplementary Table S3 (immobilisation on a monoclonal antibody). The pattern for the enrichment factors relative to wild type (EF_variant_/EF_wt_) was similar to that for paionap-5 except in the case of the K124A PAI-1 variant which, in agreement with previously published data (14), is highly important for paionap-40 binding. EF_variant_/EF_wt_ for this mutant was reduced 4-fold for paionap-40, demonstrating the ability of the branched selection-HTS assay to accurately determine the binding site of paionap-40.

**Table 2.** Sequences with a paionap-40 (SEQ ID 53156) binding site

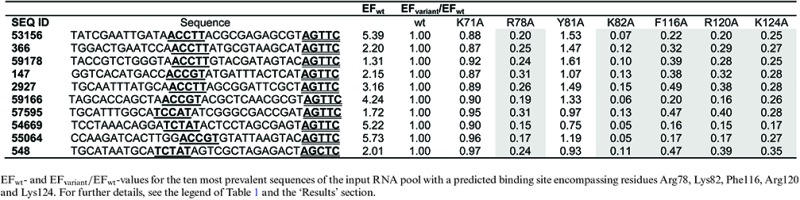										

Of the 20 206 sequences of the input RNA pool, 82 exhibited a binding pattern similar to paionap-40. The 10 most frequently observed sequences in the input RNA pool (including paionap-40) are shown in Table 2 and Supplementary Table S3. Using the MEME suite, we found that at least 74 of 82 sequences are similar to paionap-40, by the criterion of having two conserved motifs, (A/T)CC(A/G/T)T and AGTTC (Supplementary Figure S2; underlined in Table 2 and Supplementary Table S3). Hence, there is a clear correlation between sequence and binding site for paionap-40-like aptamers as well, in support of the results and the predictive power of the assay.

### Prediction of target conformation specificity and inhibitory activity towards target - natural ligand interaction

We next investigated the ability of the branched selection-HTS procedure to reveal information about differential affinity of the aptamers to different conformations of PAI-1. The results for paionap-5 and -40 are given in Table 3 after one round of parallel selection of the input pool for active and latent PAI-1, immobilised on the microtiter plate using a monoclonal antibody. In the case of paionap-40, but not paionap-5, the enrichment factor for latent PAI-1 was reduced extensively compared to that for active PAI-1. Similar results were obtained when a polyclonal antibody was used for PAI-1 immobilisation (data not shown). Hence, the data indicate that paionap-5 binds both forms of PAI-1 equally well while the affinity of paionap-40 for latent PAI-1 is lower than that for active PAI-1. This is in perfect agreement with previous biochemical data (14). The same binding pattern was observed with all paionap-5 and -40-like variants (data not shown).

**Table 3.** Binding of paionap-5 and -40 to PAI-1 conformations and the PAI-1:vitronectin complex

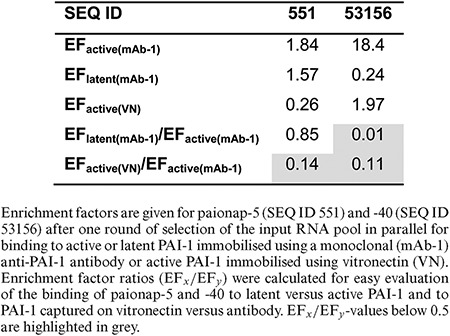										

As shown in Figure 3, the binding sites of paionap-5 and -40 on PAI-1 overlap with the binding site of the N-terminal somatomedin-B domain of vitronectin. This correlates well with the results of biochemical assays demonstrating inhibitory activity of the two aptamers towards the PAI-1-vitronectin interaction (14). As shown in Table 3, the enrichment of both paionap-5 and -40 was reduced for PAI-1 immobilised via vitronectin on the microtiter plate compared to PAI-1 displayed using a monoclonal antibody. Hence, these results demonstrate that after a single round of branched selection and HTS analysis it is possible to determine the target conformation specificities of the aptamers in a SELEX pool as well as their inhibitory activities towards target – natural ligand interactions.

### Identification of secondary structure features for paionap-5- and -40-like aptamers

**Figure 4.**
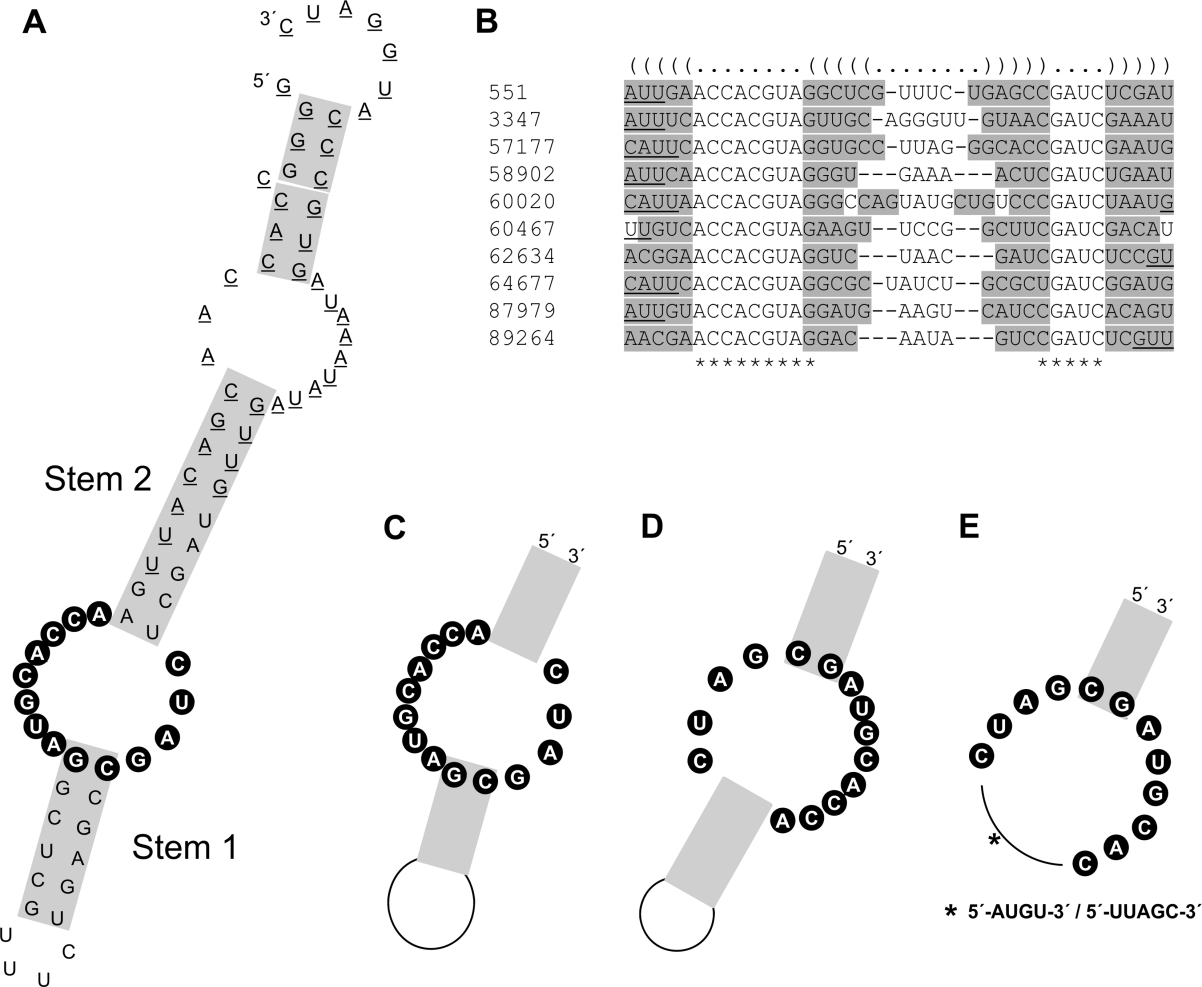
Secondary structure prediction for paionap-5 variants. (**A**) The predicted secondary structure for paionap-5 (SEQ ID 551), generated by RNAshapes. Grey boxes denote helical regions, constant flanking regions are underlined, and bases with at least 90% conservation are shown with black spheres. (**B**) Ten paionap-5 related sequences with high structural and sequence similarity based on RNAshapes predictions were aligned using LocARNA. The average secondary structure is indicated in dot-bracket notation (top). Conserved bases in the random region are indicated by an asterisk (bottom). Bases in individual sequences predicted to undergo Watson–Crick base-pairing are highlighted in grey. Most of the constant regions were omitted for clarity as these regions were invariant. Remaining constant region bases have been marked by underlining. (**C**) Consensus structure and regions of high sequence conservation based on the LocARNA alignment. (**D** and **E**) Consensus structures of other paionap-5 related families with the same binding site.

Having identified a large number of paionap-5 and -40 related sequences with similar binding characteristics, we searched for common structural features among them. We initially used the program RNAshapes (24) to predict minimum free energy secondary structures for the 97 and 74 paionap-5 and -40 like sequences, respectively. Interestingly, the position of the conserved motifs, (A/G)(C/T)CACGTAG and CGATC in the RNA secondary structure, varies in paionap-5-like sequences (Table 1 and Supplementary Table S2). Accordingly, the predicted secondary structures let us to suggest the existence of distinct structural subgroups of sequences. The predicted secondary structure for paionap-5 (SEQ ID 551) places the two conserved motif sequences in an internal loop (Figure 4A). Ten of the 97 paionap-5 variants exhibited an internal loop arrangement similar to paionap-5. Using the LocARNA alignment and folding tool (23) a common consensus structure was obtained (Figure 4B and C). A high degree of covariance was found in the predicted stem regions on both sides of the internal loop, supporting the stem-loop structure. However, no conserved elements were found in the distal loop of paionap-5 sequences, implying that this loop may be less important for binding to PAI-1. Consensus structures of other groups of paionap-5-like sequences included structures with a reversed orientation of the conserved motifs in the internal loop (Figure 4D) and structures in which the two motifs were present in a hairpin structure (Figure 4E). No covariance was detected between the sequences of the internal or hairpin loops in agreement with the possibility that these motifs could be engaged in other interactions than normal Watson–Crick base pairing (data not shown).

**Figure 5.**
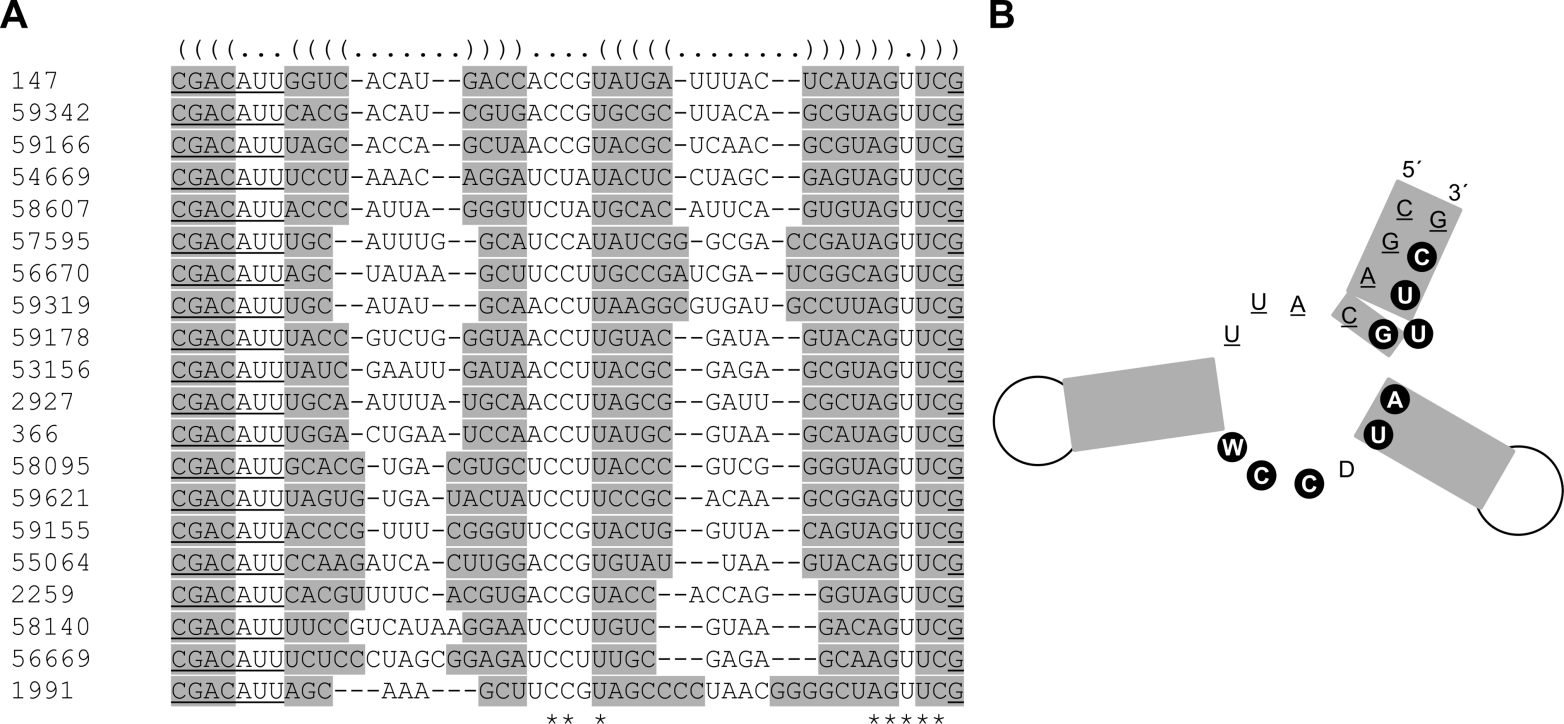
Secondary structure prediction for paionap-40 variants. (**A**) Twenty sequences with structural and sequence similarities to paionap-40 (SEQ ID 53156) were aligned using LocARNA. (**B**) The predicted average structure for paionap-40 variants based on the LocARNA alignment. See legend to Figure 4 for details. W denotes A or U, and D denotes G or T or A.

A similar analysis of the structural features for paionap-40 was performed. On the basis of the secondary structure predictions by RNAshapes, no major structural differences were observed for the 74 paionap-40-like variants. LocARNA secondary structure prediction based on alignment of sequences suggested that all sequences form a three-way helical junction in which the existence of two distal stems could be confirmed by covariance (Figure 5A and B). The proximal stem was formed by base pairing of the 5′-constant region with part of the highly conserved motif in the 3′-end of the random region while incorporating a bulge in the stem. The position of the second conserved motif suggests an important role for the proximal base pair of the second distal stem and the adjacent internal loop sequence whereas no sequence conservation was found in the opposite part of the internal loop and in the distal hairpin loops.

The consensus structures obtained suggest that PAI-1 binding could be retained by partially deleted versions of paionap-5 and -40. We reduced the constant regions of the aptamers according to the consensus to generate 46- and 49-nucleotide versions of paionap-5 and -40, respectively (P5.46 and P40.49; see figure text Supplementary Figure S3A-B). The PAI-1 binding profile for wild type and alanine mutants of PAI-1 was retained by the truncated aptamers (Supplementary Figure S3A-B) compared with the full-length versions (data not shown).

### Evaluation of the PAI-1 mutant specificity for the top 1000 most abundant sequences of the input RNA pool

**Figure 6.**
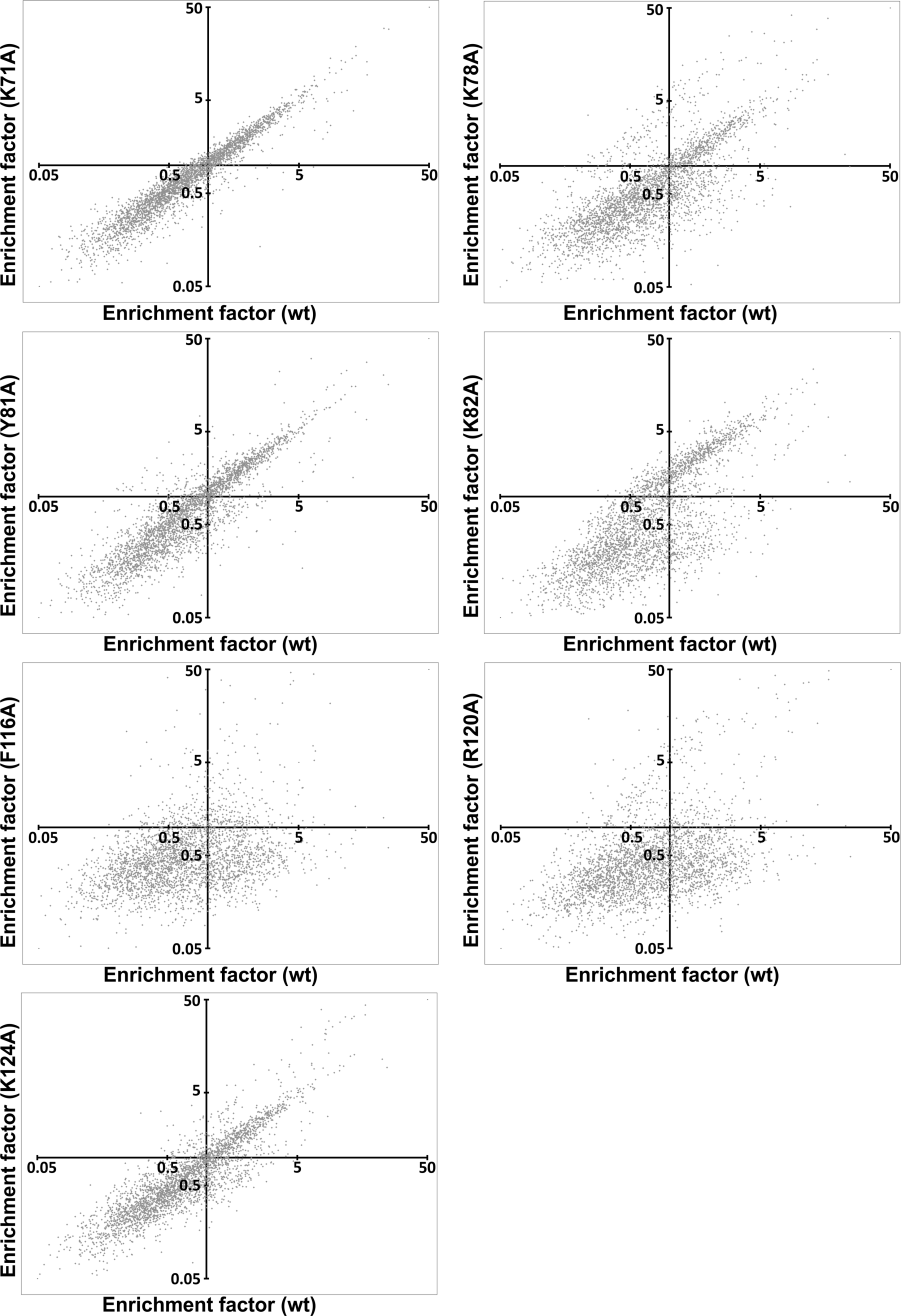
Evaluation of the PAI-1 mutant specificity of the thousand most prevalent sequences of the input RNA pool. For each single-residue alanine mutant used in the HTS-microplate assay a plot of enrichment factors for wild type (EF_wt_; x-axis) versus mutant (EF_variant_; y-axis) is shown. The x-axis crosses the y-axis at EF_variant_ = 1 and the y-axis crosses the x-axis at EF_wt_ = 1.

In order to investigate the binding sites for selected aptamers of the input RNA pool on a more global scale, we plotted the EFs upon selection with individual PAI-1 mutants for the thousand most prevalent sequences of the input RNA pool versus the EF for wild type PAI-1 (Figure 6). For the analysis we focused on the data obtained with PAI-1 immobilised via polyclonal antibody, but similar results were found with immobilisation via the monoclonal antibody (data not shown). The linear correlation, with a slope of 1, found between K71A and wild type PAI-1 indicates that the K71A mutation is irrelevant for the enrichment and therefore the binding of the majority of the sequences. In contrast, many aptamers selected against PAI-1 mutants R78A, Y81A, K82A and K124A, but most pronounced for F116A and R120A, clearly have enrichment factors different from that obtained with wild type. When we analysed the sequences with the highest wild type PAI-1 enrichment factors (the 446 sequences with EF_wt_ > 1), ∼60% of the sequences had a more than two-fold lower enrichment factor when selecting with either F116A and/or R120A. This indicates that a large proportion of aptamers are dependent on Phe116 and/or Arg120 for binding. For only ∼25% of the sequences with EF_wt_ > 1, the enrichment factors were not reduced >2-fold for any of the seven single-residue alanine mutants (data not shown). We therefore conclude that the binding area for paionap-5 and -40 must be a hotspot for a large fraction of the enriched aptamer sequences. Interestingly, a selection for aptamers against PAI-1 by another group resulted in aptamers binding to the vitronectin binding area (26).

Using a 2-fold reduction in enrichment factor as the threshold for including a residue in the binding site, we inspected the 60% of sequences with EF_wt_ > 1 and a low EF_F116A_ and/or EF_R120A_ (<0.5). Interestingly, apart from binding profiles similar to paionap-5 (R78-K82-F116-R120) and paionap-40 (R78-K82-F116-R120-K124), alternative binding profiles were also found, including R78-F116-R120, K82-F116-R120, R78-Y81-K82-F116-R120, K71-R78-Y81-K82-F116-R120 and F116-R120 (Supplementary Table S4).). We used the MEME suite to search for conserved motifs in the latter group of aptamer sequences, which was the most abundant group. We found that nearly all exhibited two common motifs, GCCGTA(G/T) and (C/T)GATC (Supplementary Figure S4). These motifs resemble the ones found in the paionap-5 variants and secondary structure predictions also localise them in a similar but slightly shorter internal loop arrangement (data not shown). Likewise, most of the sequences with the other alternative binding site patterns contained variants of the paionap-5 motifs (Supplementary Table S4). The results suggest that the paionap-5-like interactions with Phe116 and Arg120 are conserved and central elements in recognition of PAI-1 by many aptamers. However, the exact binding mode appears to vary among the paionap-5 variants.

### Finding aptamers with properties unrelated to those of paionap-5 and -40

**Figure 7.**
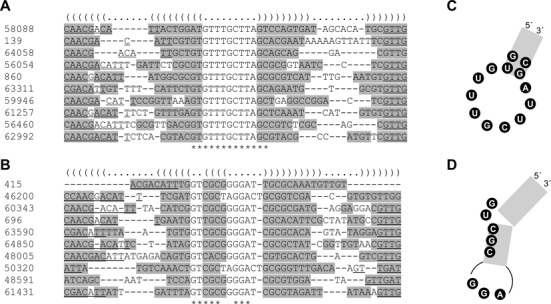
Secondary structure prediction for RNA variants binding to the PAI-1:vitronectin complex. LocARNA alignment of the ten variants of SEQ ID 58088 (**A**) and SEQ ID 415 (**B**) after one round of selection for binding to the PAI-1:vitronectin complex. (**C** and **D**) The predicted secondary structures for SEQ ID 58088 and SEQ ID 415, respectively, based on the LocARNA alignment. See the legend to Figure 4 for details.

Having validated our approach for large-scale aptamer profiling using paionap-5 and -40, we next searched the input RNA pool for sequences with unrelated properties. As both paionap-5 and -40 compete for binding to PAI-1 with vitronectin, we decided to look for sequences with the ability to bind the PAI-1:vitronectin complex. We therefore sorted the input RNA pool sequences according to enrichment factors (highest to lowest) upon one round of selection against the PAI-1:vitronectin complex. The 1000 most enriched sequences had enrichment factors between ∼7 and ∼700. A search for conserved motifs showed that the ones with highest enrichment factors could be ordered into two dominating groups with extensive sequence conservation (Supplementary Figure S5). Ten sequences from each group were aligned using LocARNA to allow the identification of structural features (Figure 7). One family of sequences was found to adopt hairpins with high degree of conservation in the distal loop sequence (Figure 7A and C). The other family of sequences adopt hairpins with sequence conservation in the distal loop as well, but also contain a conserved 2-nucleotide bulge in the stem (Figure 7B and D).

To confirm the binding properties of these sequence families one member with a high enrichment factor from each family was produced by RNA transcription (SEQ ID 415, EF_PAI-1:VN_ = 675 and SEQ ID 58088, EF_PAI-1:VN_ = 470). The binding of the two aptamers to PAI-1 and the PAI-1:vitronectin complex was investigated by SPR analysis. In one setup, the aptamers were biotinylated and captured on streptavidin sensor surfaces (Supplementary Figure S6). Passing PAI-1 with and without a 10-fold excess of vitronectin over the sensor surfaces, we demonstrated a lack of ability of vitronectin to compete with the binding of SEQ ID 415 and SEQ ID 58088 to PAI-1. We confirmed this result using another setup in which PAI-1 was captured on SPR sensor surfaces coated with vitronectin. As expected, SEQ ID 415 and SEQ ID 58088 (but not paionap-5 and -40) were able to bind PAI-1 immobilised on vitronectin. Interestingly, SEQ ID 415 was found only 22 times in the input pool among 1.58 × 10^6^ sequence reads, corresponding to the ∼11 000th most abundant sequence (Figure 2A), but after one round of selection for the PAI-1:VN complex it was the third most abundant sequence. SEQ ID 58088 was detected only 2 times in the input pool corresponding to the ∼55 000th most abundant sequence but became the 230th after selection. The two sequences were therefore highly underrepresented before the branched selection step. The results suggest that sequences with the ability to bind PAI-1 may even exist below the detection limit of our sequencing analysis.

## DISCUSSION

With the large number of candidate aptamers emerging from HTS analyses of SELEX pools, novel strategies are needed for rapid and simple high-throughput sorting or characterisation of the sequences (1). It has already been demonstrated that direct assessment of round-to-round sequence enrichment during early rounds of SELEX can facilitate the identification of aptamers with high affinity in complex pools (11). Here, we present a strategy to quickly gain an overview of the properties of thousands of aptamer candidates simultaneously in terms of binding sites, target conformation specificity and functional properties. This has to our knowledge not been demonstrated before. In addition, we were also able to generate structure-function information for individual groups of related aptamers using a pipeline of already available web tools such as RNAshapes, the MEME suite and LocARNA.

Interesting examples of other high-throughput aptamer or aptamer candidate characterisation strategies was reviewed recently (1) and mainly involves microarray- and Illumina platform-based approaches (13,27–29). For example, Cho *et al*. (13) previously presented an array-based screening approach in which high copy number sequences, identified from HTS analysis of DNA pools after selection for angiopoietin-2, were synthesised on an microarray chip followed by monitoring the binding of fluorescently-labelled protein targets. Aptamer target affinity (*K_D_*) and specificity for angiopoietin-2 could be determined, as well as performance in serum. In addition, analysis of affinities for consensus group sequences enabled identification of specific sequence elements important for binding. In Tome *et al*. (29) randomised RNA aptamers for Drosophila negative elongation factor E and green fluorescent protein were produced on the Illumina platform and probed for target affinity to gain insights into RNA regions of importance for RNA-protein interaction.

Importantly, the procedure presented here, directly assesses the aptamer candidates present in the SELEX pool without the need to choose subpopulation of sequences, manufacture individual aptamers or apply advanced instrumentation or methods. The procedure simply requires a single round of branched selection on a pool of RNA enriched for target-binding followed by HTS, and can easily be tailored to investigate a wider range of aptamer properties.

The input RNA pool used in our study had previously been enriched for PAI-1 binding over 5 rounds of standard selection. At this point, we used HTS to analyse the composition of the pool and identified 69 945 unique sequences among 1.58 × 10^6^ reads. The number of unique sequences obtained naturally depends on the total number of sequence reads and the diversity of the pool. No sequence comprised more than approximately 0.6% of the pool. The chance of picking the previously identified aptamers paionap-5 and -40 from the RNA pool by standard procedures would have been 0.41% and 0.069%, respectively. Hence, after 5 rounds of selection the pool was highly diverse and the selection of any specific PAI-1 binding sequence for further analysis would not have been feasible.

To identify the binding sites of aptamers, we screened the input RNA pool, using a microtiter plate setup, for binding to either wild type PAI-1 or a panel of single-residue alanine mutants of PAI-1 and monitored the subsequent changes of sequence frequencies in the pool (the enrichment factors) by HTS analysis. Making the assumption that only target binding contributes to the selection of aptamers, we hypothesised that for each sequence the enrichment factors would correlate with PAI-1 variant affinity. The single-residue alanine mutants was a panel of PAI-1 variants for which paionap-5 and -40 had previously been shown to exhibit reduced affinity (14). Without prior knowledge of the binding site, we would suggest residues in and around positive patches on the target or variants with domain truncations as candidates for analysis. For both paionap-5 and -40, there was an excellent agreement between the previous biochemical data and the branched selection-HTS data. To support the results we first estimated the experimental variation when preparing samples for HTS analysis. Importantly, only minor variations were observed in independently prepared HTS data sets for the input RNA pool, even after an additional PCR-amplification of the DNA template. Secondly, since the PAI-1 handle, used to immobilise the protein during selection, potentially could influence the result, we carried out the microtiter plate selection assay using both a polyclonal and a monoclonal antibody for PAI-1 immobilisation and obtained the same result. Thirdly, we searched the input RNA for sequences with predicted binding sites similar to those for paionap-5 and -40, and identified primarily paionap-5 and -40 variants. When scanning the HTS data, we generally chose a 2-fold reduction of enrichment factors for an alanine mutant relative to PAI-1 wild type as our threshold for including a particular residue in the binding site. The chosen threshold, and the requirement that data had to coincide independently of PAI-1 immobilisation mode, are of course optional parameters, which can be optimised or excluded, respectively, and which determine the reverse relationship between stringency and the number of output sequences. Importantly, the threshold must be considered relative to the experimental variability inferred by sample preparation and HTS analysis. Interestingly, the smaller reduction in enrichment factor observed with paionap-5 for K124A did correlate with a slight increase in the dissociation rate for the complex between the mutant and the aptamer. However, care should generally be taken when interpreting minor differences in the enrichment factors given the standard deviations for the procedure, small variations in the content of active versus latent protein in the PAI-1 preparations, etc.

Previously, paionap-5 and -40 were investigated by RNase footprinting and phosphate modification interference analysis to determine aptamer regions important for PAI-1 binding (14). Although the resolution of the analysis was limited by the nuclease-resistance of the 2′-fluoropyrimidines in the aptamers, the predicted internal loop and the stem of the distal hairpin in paionap-5 were found to be the sites most likely mediating direct interaction with PAI-1. For paionap-40, the multibranched loop was suggested to be central for direct binding to PAI-1 (14). The comparison of aptamer variants in the present study confirmed the predicted secondary structure of the aptamers.

The aim of a SELEX experiment usually is to identify aptamers with highest affinity for a given target. However, sequencing of a single pool at a late stage of the selection will mainly contain target binders, which may not be the ones with highest affinity, but which may dominate because they contain less complex binding domains and are proportionally more common in the starting pool. In our case, the input pool was a result of five rounds of selection in which the PAI-1 was present at a high concentration and in excess over binding RNA. For the input pool sequences we did not find a correlation between abundance and affinity for PAI-1, indicating that the selection conditions up to this point favoured binding but not necessarily high affinity. The round-to-round enrichment factors during target selection have been found to be a better indicator of high affinity binding of aptamers (11). However, the enrichment factors will only reflect the affinity if this is the main selection criteria. Unlike the first five rounds of selection, the branched selection step involves immobilisation of relatively low amounts of PAI-1 in a microtiter plate. Hence, the more competitive conditions of this selection step should favour high affinity. It is important to note, however, that parameters such as sequence abundance in the pool and the mode of target presentation may also affect affinity and relative enrichment factors. When inspecting the results from the branched selection, the enrichment factors for paionap-5 and -40 variants, after one round of wild type PAI-1 selection, could indicate that paionap-40 is among the best binding candidates. Among sequences with predicted binding sites similar to paionap-5, 20–30 sequences had higher EF_wt_ compared with paionap-5, for example SEQ ID 59177 (Table 1 and Supplementary Table S2). Although conserved motifs were found in nearly all paionap-5 and -40 variants, enrichment factors could vary almost one order of magnitude, suggesting that RNA regions outside these highly conserved areas may contribute to PAI-1 affinity or RNA structuring.

While paionap-5 and -40 have similar binding sites, only paionap-5 is able to bind both the active and latent conformation of PAI-1, whereas paionap-40 only binds the active form (14). The reason was attributed to the importance of Lys124 for paionap-40 binding to PAI-1, which unlike the other residues important for binding, is relocated upon active-to-latent transition (14). In the current study, we demonstrated that the branched selection-HTS assay can be used to decipher target conformational specificity of aptamers in a pool. As the binding site of the aptamers in PAI-1 overlaps with the binding site of vitronectin (Figure 3), the compatibility of aptamer binding to the PAI-1:vitronectin complex was also investigated as another predictor of aptamer interference with this interaction. The result demonstrates that the procedure can be designed to assist in the discovery of aptamers with particular functional properties.

During the analysis of the data, we noticed a tendency for the majority of sequences in the input RNA pool to have more than two-fold reduced enrichment factors for at least one or several of the applied single-residue alanine PAI-1 mutants compared to wild type PAI-1. We therefore carried out a global analysis for the 1000 most prevalent RNA sequences in the pool in terms of their binding sites in PAI-1 and found that the majority probably recognise the same region as paionap-5 and -40. This could indicate that the area is a particular hotspot for high affinity binding of aptamers to PAI-1. We also found that most of the sequences binding in this region have sequence and secondary structural features similar to paionap-5 and -40. These findings suggest that overall there might be a rather limited number of three-dimensional structures that RNA sequences can adopt to mediate high affinity binding to this region of PAI-1. Interestingly, paionap-5 related variants not only exhibit a large degree of variation in sequence and affinity (as predicted from enrichment factors), but the HTS data also suggest that there is diversity in the specific recognition of PAI-1 by different variants.

The input RNA pool appears to be dominated by a limited number of aptamer families sharing similar functional characteristics. We decided to investigate the possibility of finding aptamers in the pool with characteristics different from those of paionap-5 and -40. As most PAI-1 *in vivo* is presumed to be in complex with its high affinity ligand vitronectin, aptamers capable of binding to the PAI-1:vitronectin complex could be of interest. Searching the input RNA pool for aptamers with high enrichment for the PAI-1:vitronectin complex allowed us to identify underrepresented aptamers in the pool with this property.

Hence, with a multitude of conditions screened, branched selection combined with HTS analysis may potentially allow a global analysis of sequences from SELEX pools for aptamer affinities and specificities, structural features, RNA-protein interactions, and functional properties.

## Supplementary Material

SUPPLEMENTARY DATA
