# Supplementary material for: Characterisation of aptamer–target interactions by branched selection and high-throughput sequencing of SELEX pools
Source: Nucleic Acids Res. 2015 Jul 10;43(21):e139. doi: 10.1093/nar/gkv700 (PMC4666376; doi:10.1093/nar/gkv700)
Supplement: SUPPLEMENTARY DATA [file supp_43_21_e139__index.html]

Characterisation of aptamer–target interactions by branched selection and high-throughput sequencing of SELEX pools — Characterisation of aptamer–target interactions by branched selection and high-throughput sequencing of SELEX pools — SUPPLEMENTARY DATA 

# Characterisation of aptamer–target interactions by branched selection and high-throughput sequencing of SELEX pools

## SUPPLEMENTARY DATA

- SUPPLEMENTARY DATA
