# Supplementary material for: Characterisation of aptamer–target interactions by branched selection and high-throughput sequencing of SELEX pools
Source: Nucleic Acids Res. 2015 Jul 10;43(21):e139. doi: 10.1093/nar/gkv700 (PMC4666376; doi:10.1093/nar/gkv700)

Supplementary Information for "Characterisation of aptamer-target interactions by branched selection and high-throughput sequencing of SELEX pools".

Table S1. The 50 most abundant unique sequences ranked according to their frequency in the input pool sample. From the left, the sequence identification number (SEQ ID) and the corresponding selected sequence (shown as DNA). The number of reads for each sequence was counted and converted to "Percentage of pool" (%) by dividing with the total number of sequence reads. Also shown are the HTS data obtained independently two times for the original RNA pool of the PAI-1 aptamer selection experiment after five rounds of selection (Original #1 and #2). In addition, percentage of pool and enrichment factor values (EF_{wt}) after one round of selection for wild type recombinant PAI-1 (Wild type 1R) immobilised using polyclonal anti-PAI-1 antibody are included to demonstrate data conversion (see results text).

		**Sequence read number**			**Percentage of pool (%)**				**EF_{wt}**
**SEQ ID**	**Sequence**	**Input pool**	**Original #1**	**Original #2**	**Input pool**	**Original #1**	**Original #2**	**Wild type 1R**	**Wild type 1R**
**2202**	CAGGCCGTAGGATGTTATATCCGATCCTGATCTTC	8975	9954	7086	0.567	0.574	0.580	0.796	1.40
**5378**	GTGACCCACCATTATATGGTGACTAGTAGCGTGTT	8330	8971	6671	0.527	0.517	0.546	0.091	0.17
**47414**	TTGTAGGTGTCAACTACCGACCGTCGTTTGCATTA	6772	7487	5529	0.428	0.432	0.452	0.533	1.24
**551**	GAACCACGTAGGCTCGTTTCTGAGCCGATCTCGAT	6410	6611	4658	0.405	0.381	0.381	0.655	1.62
**88284**	GCGCGAAGCGCTTACGCGTGTTGATCGAATGTT	6296	7846	6047	0.398	0.453	0.495	0.557	1.40
**1016**	ACCATGTAGGCGTAATTTCACGCTGACCAAGTGTT	4006	4239	3062	0.253	0.244	0.251	0.302	1.19
**13**	TGCGTAAGCATCCTTCGATGTACCACATCGAGATC	3748	3925	2730	0.237	0.226	0.223	0.247	1.04
**85382**	GACCCGTAGTGTCTTACTACGACTTGTAGATGTT	3548	3769	2668	0.224	0.217	0.218	0.111	0.50
**49347**	TCGCAGCTTCGTAGTACAGGCGCAAGTTGACACAT	3502	3551	2510	0.221	0.205	0.205	0.326	1.47
**1401**	CAACGTGCACTGTAGCTGTACAAAGCGACCAAC	3438	3283	2466	0.217	0.189	0.202	1.354	6.23
**366**	TGGACTGAATCCAACCTTATGCGTAAGCATAGTTC	3177	3403	2189	0.201	0.196	0.179	0.441	2.20
**85137**	GTAGACTTGTCAACGGTCGACTGTCGATACATGT	3173	3377	2340	0.201	0.195	0.191	0.112	0.56
**23**	GTAGCATAATCAACCTGCGACTGTCGTGTGATAAG	2958	2971	2201	0.187	0.171	0.180	0.191	1.02
**59131**	CGACCAGACGACAAGTCGTCGCACCGTAGAGTGTA	2937	3063	2284	0.186	0.177	0.187	0.029	0.15
**59178**	TACCGTCTGGGTAACCTTGTACGATAGTACAGTTC	2776	3055	2008	0.176	0.176	0.164	0.230	1.31
**3627**	GATCACTAAGGTTAGACCGGAGTGCCGTAGAATGC	2776	2925	2027	0.176	0.169	0.166	0.154	0.88
**301**	GAGCTACGGCTAAGCCGCTATAGTCTTCAGGAATC	2669	3044	2132	0.169	0.176	0.174	0.056	0.33
**467**	TCCGTGAGGATCTATGTTGTGGAAAACAACAGTTC	2599	2901	1914	0.164	0.167	0.157	0.180	1.09

**881**	TGGCGAGATGCCATCTTTACCTTAATAGGTAGTTC	2581	2946	1917	0.163	0.170	0.157	0.452	2.77
**317**	GAACGTAAGGAGAGTGCGCTTACCGCACAATGTTT	2549	2724	1921	0.161	0.157	0.157	0.069	0.43
**617**	GGTAATGGTGTGAATTAACCTTAAGCCTATTGGTT	2437	2866	1726	0.154	0.165	0.141	0.034	0.22
**56479**	ATACAGCCGATCATGTCACGTAGGCTGTGATTAAC	2385	2632	1925	0.151	0.152	0.157	0.087	0.58
**87979**	GTACCACGTAGGATGAAGTCATCCGATCACAGT	2358	2279	1691	0.149	0.131	0.138	0.129	0.87
**59192**	CTCGAGAGAGACCTTCCTCGTTATACGGGGAGTTC	2325	2800	1925	0.147	0.161	0.157	0.082	0.56
**23284**	TGCGTAAGCAACCTTGGTGTTATATTCACCAGATC	2266	2448	1726	0.143	0.141	0.141	0.090	0.63
**498**	GCGCTACGATCAAAACACGTAGTAGTGCAACGAAT	2259	2279	1596	0.143	0.131	0.131	0.041	0.29
**903**	GACCCATCTCGCGTTTCCGGGATGACTAGTAGAAT	2218	2404	1739	0.140	0.139	0.142	0.079	0.56
**59189**	CAGCGATGTAGTGCACATTGCACGACCATGATGGA	2205	2186	1654	0.139	0.126	0.135	0.073	0.53
**89**	CGTGTTGTATCTGTTATACAGCATGTTATTTGTGC	2193	2586	1683	0.139	0.149	0.138	0.516	3.72
**921**	GCACCTGTAGGCTAGGTCACTGGCTGACTGCAATT	2158	2348	1674	0.136	0.135	0.137	0.064	0.47
**3771**	ATTTTCCGGTCGTTTAGGTGTTCAACACTCCGGAT	2148	2834	1662	0.136	0.163	0.136	1.185	8.72
**1167**	GATGATCACTGTTTCGCGCAGTGCCGTATATCGAT	2061	2071	1450	0.130	0.119	0.119	0.568	4.36
**2338**	TCACACTGTAAGGCCATGTGCCGACTAGAAATTGC	2000	2057	1426	0.126	0.119	0.117	0.107	0.84
**441**	GACTCCTAGTTATACCGATTAGGACTTGTAGATGC	2000	2146	1428	0.126	0.124	0.117	0.033	0.26
**894**	ACGTAGCATGATGAGTTTCTCGTCGGCGATCAGTT	1951	2119	1491	0.123	0.122	0.122	0.121	0.98
**271**	GCCGTAGTGCAGGGTTGCTTGCATGATCAATTGTT	1914	2069	1532	0.121	0.119	0.125	0.254	2.10
**5351**	TAGGGTGCCGATCATGTCACGTAGGCATCCGATAC	1861	1923	1359	0.118	0.111	0.111	0.049	0.42
**4993**	CAACGACCAGTACGTGTGAGGGCGTACATCATGTA	1842	1896	1454	0.116	0.109	0.119	0.043	0.37
**35**	GACCCGGTCACTTATGTGGCCGATATCGTAGATGC	1815	2029	1498	0.115	0.117	0.123	0.023	0.20
**985**	CCGCTCATGATCTGTTATCATGAGTGCGTAGTACC	1758	1759	1237	0.111	0.101	0.101	0.120	1.08
**59167**	ACGTAGTTTGAGATAGTTAACTCGAACGATTAGTT	1756	2018	1363	0.111	0.116	0.112	0.174	1.57
**132**	GATCAACGATCACCATGATACACATGGATCACGTA	1749	1667	1183	0.111	0.096	0.097	0.069	0.62
**1742**	TTACTGTAGAGAAGAATTACTTCTCGACCAAATGTG	1731	2201	1391	0.109	0.127	0.114	0.060	0.55
**59384**	ACATACCGTAGGGCAGATCGCCTGACTAGTAATAC	1727	1915	1346	0.109	0.110	0.110	0.026	0.24
**318**	ATGAGCCCTATCTATCGACCGATCATGTCACGTAG	1687	1737	1247	0.107	0.100	0.102	0.147	1.38
**29**	GCTGTGCTAATCGGTCTTCCGACGCATATTCGAAT	1679	1714	1310	0.106	0.099	0.107	0.067	0.63
**59130**	TCGACTTGTAGACGATATTCGTCGACTCGAAGAGC	1679	1814	1283	0.106	0.105	0.105	0.054	0.51
**59187**	ACACTGTAGAGAATACCCAATTCTCGACTAGATGC	1657	1913	1237	0.105	0.110	0.101	0.077	0.74
**397**	AGCAGTCGTAGGCCGTAATGGCTGACCTGTATGAC	1650	1782	1446	0.104	0.103	0.118	0.071	0.68
**2091**	CCGTCCGTGTTCAGTTAACACGCATGTGTTTTGTG	1641	1651	1159	0.104	0.095	0.095	0.141	1.36

Table S2. Sequences with a paionap-5 (SEQ ID 551) binding site. EF_{wt} – and $EF_{variant}/EF_{wt}$ – values for the ten most prevalent sequences of the input RNA pool with a predicted binding site encompassing residues Arg78, Lys82, Phe116 and Arg120. For further details see the legend of Table 1 and the results section. Data was obtained with PAI-1 immobilised using monoclonal anti-PAI-1 antibody. Most prominent sequence similarities based on MEME suite analysis are highlighted using single and double underlinings.

		**EF_{wt}**	**$EF_{variant}/EF_{wt}$**							
**SEQ ID**	Sequence		wt	K71A	R78A	Y81A	K82A	F116A	R120A	K124A
**551**	GA**ACCACGTAG**GCTCGTTTCTGAGC**CGATC**TCGAT	2.66	1.00	1.17	0.14	0.84	0.16	0.09	0.09	0.78
**56479**	ATACAGC**CGATC**AT**GTCACGTAG**GCTGTGATTAAC	0.81	1.00	1.01	0.24	2.75	0.11	0.23	0.18	1.13
**87979**	GT**ACCACGTAG**GATGAAGTCATC**CGATC**ACAGT	1.37	1.00	1.07	0.13	1.18	0.16	0.14	0.12	1.07
**59189**	CA**GCGATGTAG**TGCACATTGCA**CGACC**ATGATGGA	0.44	1.00	1.10	0.42	3.99	0.26	0.45	0.34	1.52
**2338**	TC**ACACTGTAA**GGCCATGTGC**CGACT**AGAAATTGC	0.55	1.00	1.16	0.29	0.57	0.31	0.48	0.49	0.98
**5351**	TAGGGTGC**CGATC**AT**GTCACGTAG**GCATCCGATAC	0.62	1.00	1.07	0.26	2.94	0.11	0.23	0.15	1.00
**2245**	AAC**AACCCGTAG**ACGTCATCGT**CGATC**GCGTTAGT	1.35	1.00	1.15	0.28	0.86	0.27	0.28	0.21	1.06
**322**	TGCAC**ACCACGTAG**GCAAATTGC**CGATC**GTGCTAT	1.30	1.00	1.06	0.17	1.09	0.21	0.23	0.16	0.97
**435**	**GTACTGTAG**TCTTGATTTTAAGA**CGACC**AAATGTA	1.02	1.00	1.23	0.17	0.52	0.33	0.30	0.38	0.77
**59177**	TGTCAAC**CGATC**ACGCTTATAGAGCG**ACCACGTAG**	5.14	1.00	1.15	0.12	0.78	0.11	0.16	0.11	0.63

Table S3. **Sequences with a paionap-40 (SEQ ID 53156) binding site.** EF_{wt} – and $EF_{variant}/EF_{wt}$ – values for the ten most prevalent sequences of the input RNA pool with a predicted binding site encompassing residues Arg78, Lys82, Phe116, Arg120 and Lys124. For further details see the legend of Table 1 and the results section. Data was obtained with PAI-1 immobilised using monoclonal anti-PAI-1 antibody. Most prominent sequence similarities based on MEME suite analysis are highlighted using single and double underlinings.

		**EF_{wt}**	**$EF_{variant}/EF_{wt}$**							
**SEQ ID**	Sequence		wt	K71A	R78A	Y81A	K82A	F116A	R120A	K124A
**53156**	TATCGAATTGATA**ACCTT**ACGCGAGAGCGT**AGTTC**	9.69	1.00	0.77	0.09	1.42	0.03	0.11	0.09	0.13
**366**	TGGACTGAATCCA**ACCTT**ATGCGTAAGCAT**AGTTC**	3.55	1.00	0.73	0.11	1.44	0.05	0.16	0.16	0.18
**59178**	TACCGTCTGGGTA**ACCTT**GTACGATAGTAC**AGTTC**	1.53	1.00	0.74	0.13	1.62	0.06	0.24	0.16	0.23
**147**	GGTCACATGACC**ACCGT**ATGATTTACTCAT**AGTTC**	2.36	1.00	0.71	0.19	1.57	0.09	0.25	0.22	0.29
**2927**	TGCAATTTATGCA**ACCTT**AGCGGATTCGCT**AGTTC**	3.88	1.00	0.66	0.16	1.55	0.09	0.26	0.21	0.17
**59166**	TAGCACCAGCTA**ACCGT**ACGCTCAACGCGT**AGTTC**	5.48	1.00	0.72	0.08	1.44	0.03	0.10	0.11	0.12
**57595**	TGCATTTGGCA**TCCAT**ATCGGGCGACCGAT**AGTTC**	2.86	1.00	0.74	0.14	1.02	0.05	0.21	0.32	0.21
**54669**	TCCTAAACAGGA**TCTAT**ACTCCTAGCGAGT**AGTTC**	3.43	1.00	0.72	0.14	1.12	0.05	0.22	0.28	0.34
**55064**	CCAAGATCACTTGG**ACCGT**GTATTAAGTAC**AGTTC**	4.62	1.00	0.77	0.13	1.42	0.05	0.14	0.18	0.26
**548**	TGCATAATGCA**TCTAT**AGTCGCTAGAGACT**AGCTC**	1.69	1.00	0.71	0.23	0.99	0.12	0.44	0.37	0.41

Table S4. Paionap-5 related sequences with alternative binding sites as predicted from the branched selection-HTS assay. The ten most prevalent sequences in the input RNA pool with the following binding sites are shown: (**A**) R78-F116-R120, (**B**) K82-F116-R120, (**C**) R78-Y81-K82-F116-R120, (**D**) K71-R78-Y81-K82-F116-R120 and (**E**) F116-R120. Most prominent sequence similarities based on MEME suite analysis are highlighted using single and double underlinings.

A

		**EF_{wt}**	**$EF_{variant}/EF_{wt}$**							
**SEQ ID**	Sequence		wt	K71A	R78A	Y81A	K82A	F116A	R120A	K124A
**59350**	CTACGT**CGTAG**TCGCTTAGCGA**TGACCG**TTAGAT	0.74	1.00	1.01	0.32	0.90	1.24	0.36	0.30	0.73
**305**	GT**CGTAG**GCGTCATATTATACTTACGC**TGACCA**AT	2.09	1.00	1.19	0.39	1.19	2.66	0.23	0.20	1.03
**59310**	GCAT**CGTAG**GCATATAGC**TGACTG**CAATTTAGGAC	2.00	1.00	1.18	0.39	0.67	1.66	0.18	0.15	0.60
**938**	TGACAT**CGTAG**GCAAAAAAGC**TGACTG**TTCAGATT	2.86	1.00	1.09	0.33	0.69	1.09	0.26	0.20	0.74
**89744**	A**CGTAG**CTGGGTTTCACCAG**CGATTA**GTT	0.70	1.00	0.95	0.45	0.53	0.59	0.42	0.22	0.93
**243**	GT**CGTAG**GACGTGAGTGTC**TGACCG**ATGTAGTTGG	0.65	1.00	1.05	0.42	1.27	2.26	0.20	0.20	0.97
**58760**	GT**CGTAG**GCAGATAGATTATATCCGC**TGACCA**GT	2.25	1.00	1.06	0.34	0.99	2.24	0.17	0.17	0.97
**56746**	GT**CGTAG**CCGGACTCCACCGG**TGACCA**ATGAGC	0.57	1.00	1.21	0.47	1.15	2.87	0.23	0.21	0.88
**84077**	TACGCAC**CGTAG**ACTTAATAGT**CGATTA**GTACAT	1.19	1.00	1.06	0.34	0.58	0.58	0.34	0.33	0.60
**58617**	GT**CGTAG**GCAAGTGTACGAATCTTTGC**TGACCA**GT	2.67	1.00	1.11	0.44	1.01	2.38	0.19	0.16	0.99

B

		**EF_{wt}**	**$EF_{variant}/EF_{wt}$**							
**SEQ ID**	Sequence		wt	K71A	R78A	Y81A	K82A	F116A	R120A	K124A
**262**	TGTCAACATA**ACGTAG**AGCAATTACAGCT**CGATCA**	3.13	1.00	0.99	1.05	1.50	0.27	0.33	0.29	1.17
**59137**	TCATA**ACGTAG**ACTGTCTGAGT**CGATCA**GAGTGTT	1.68	1.00	1.04	0.95	1.70	0.28	0.33	0.29	1.23
**58835**	ATCGACCATA**ACGTAG**CGGGCTTTCCCG**TGATCA**G	0.91	1.00	0.93	1.11	1.49	0.21	0.18	0.27	1.10
**58762**	**ACGTAG**TTGCCTAATAAAGGTAG**CGATTT**GGATTC	1.28	1.00	1.16	0.87	0.85	0.17	0.31	0.34	1.89
**59940**	CGTA**ACGTAG**GGAGTATTCC**CGATCA**GAATGTACA	0.36	1.00	1.08	1.26	2.31	0.43	0.41	0.36	1.12
**60130**	**ACGTAG**GTAGCATTTATATGCTTGAC**CGATCA**ATC	1.07	1.00	0.87	0.58	0.52	0.27	0.40	0.28	0.77
**59868**	CGGGACT**ACGTAG**GCGGATTCGC**CGATTA**CCCGTT	0.70	1.00	1.01	0.71	0.94	0.48	0.34	0.22	1.17
**60345**	CACACA**ACGTAG**GCTCGTGAGC**CGATCA**GTGATTA	0.81	1.00	0.93	0.88	1.54	0.26	0.25	0.28	0.92
**59853**	CCTGCA**ACGTAG**GCGCGTAAGCTC**CGATCA**AGGAT	0.53	1.00	0.97	1.17	1.62	0.42	0.43	0.25	1.41
**58979**	T**TGATCA**ACACA**ACGTAG**GTCTATGCGACCGATA	2.02	1.00	0.92	1.14	1.40	0.29	0.27	0.39	1.08

C

		**EF_{wt}**	**$EF_{variant}/EF_{wt}$**							
**SEQ ID**	Sequence		wt	K71A	R78A	Y81A	K82A	F116A	R120A	K124A
**1016**	**ACCATGTAG**GCGTAATTTCACGCT**GACCA**AGTGTT	1.19	1.00	1.13	0.19	0.26	0.14	0.30	0.28	0.74

**1401**	CAACGT**GCACTGTAG**CTGTACAAAGC**GACCA**AC	6.23	1.00	1.15	0.08	0.42	0.15	0.07	0.18	0.51
**88166**	**ACACTGTAG**CGAGCCAGACTCGC**GACCA**AATGC	0.89	1.00	1.30	0.22	0.46	0.22	0.29	0.38	0.58
**69**	GT**GCCATGTAG**ATCTTTAGGAGATC**GACCA**GCAGT	0.57	1.00	1.02	0.29	0.39	0.29	0.37	0.36	0.69
**1028**	GACTAGACATGATGTTGCATGTC**ACCATGTAA**AAT	0.92	1.00	1.15	0.37	0.41	0.25	0.47	0.46	0.72
**59105**	G**ACCATGTAG**GACTTAAAGTCT**GACCA**CAATGTGT	0.60	1.00	1.06	0.27	0.36	0.28	0.44	0.41	0.77
**330**	G**AACTTGTAG**TACCTGTATTGGGTAT**GACCT**CAGT	1.61	1.00	1.06	0.26	0.41	0.25	0.19	0.32	0.70
**222**	C**GCACTGTAG**CTATGTGGATAGC**GACCA**GAATTTC	2.51	1.00	1.22	0.11	0.41	0.12	0.13	0.27	0.53
**272**	ACG**AACTTGTAG**GCACGAATACGCC**GACCT**CTAAT	1.25	1.00	1.11	0.31	0.43	0.29	0.22	0.31	0.57
**59143**	C**ACCACGTAG**TGAAACTTTCAC**GACTA**GAATGTAA	0.74	1.00	1.13	0.23	0.34	0.23	0.37	0.30	0.61

D

		**EF_{wt}**	**$EF_{variant}/EF_{wt}$**							
**SEQ ID**	Sequence		wt	K71A	R78A	Y81A	K82A	F116A	R120A	K124A
**17**	**AAGTTCGTCC**TCTTCCAAGAGAAG**GGGTCTA**TGCC	5.35	1.00	0.14	0.08	0.03	0.17	0.14	0.09	4.71
**59175**	**GAGTCTC**CTACACGCGTAGGT**AAGTTCGTCT**CATT	3.79	1.00	0.22	0.12	0.08	0.20	0.25	0.14	2.97
**59392**	C**TAGTCTG**GTAGCAATACCT**AAGTTCGTCT**AGAAA	0.89	1.00	0.24	0.11	0.10	0.22	0.26	0.23	2.87
**59370**	**AAGTTCGTCC**CGCATGGTACGATGC**GGGTCTA**TGC	6.47	1.00	0.15	0.12	0.06	0.13	0.21	0.13	4.16
**625**	**AAGTTCGTCC**CCGTAAGCTCTTACG**GGGTCTA**TGC	5.92	1.00	0.13	0.08	0.08	0.13	0.15	0.12	4.18
**59311**	GTTGAGCGCT**GAGTTTC**T**GCGTTAGTTC**TGTCAAT	3.37	1.00	0.30	0.27	0.14	0.36	0.33	0.30	2.03
**59277**	T**AAGTTCGTCT**CACTGACTCTGCAGT**GAGTCTA**AT	7.58	1.00	0.17	0.08	0.06	0.21	0.15	0.08	3.99
**7**	**GAGTCTA**GACAAAATTGTCTT**AAGTTCGTCT**TAAT	7.89	1.00	0.18	0.06	0.07	0.13	0.19	0.16	2.74
**56649**	A**AGTTTCGTCC**GGATCGTGCAATC**CGGTCTA**TGTG	4.02	1.00	0.20	0.21	0.10	0.30	0.29	0.18	4.04
**60065**	CT**AAGTTCGTCC**TACTTACTCTTGT**AGGTCTG**AAT	16.38	1.00	0.16	0.06	0.02	0.11	0.10	0.04	3.88

E

		**EF_{wt}**	**$EF_{variant}/EF_{wt}$**							
**SEQ ID**	Sequence		wt	K71A	R78A	Y81A	K82A	F116A	R120A	K124A
**2202**	CAG**GCCGTAG**GATGTTATATC**CGATC**CTGATCTTC	1.40	1.00	1.12	1.27	1.23	1.89	0.25	0.21	1.19
**1167**	GA**TGATC**ACTGTTTCGCGCAGT**GCCGTAT**ATCGAT	4.36	1.00	1.30	0.72	1.15	1.52	0.20	0.15	0.84
**271**	**GCCGTAG**TGCAGGGTTGCTTGCA**TGATC**AATTGTT	2.10	1.00	0.99	0.75	1.00	1.61	0.24	0.20	0.92
**57406**	**GCCGTAG**GAATTCGTCCTGAGTTC**CGATC**AATAGC	0.74	1.00	1.07	0.96	1.11	1.93	0.18	0.23	1.08
**478**	**GCCGTAG**GCGATAATACATCGC**TGATC**ATTTGTAC	2.34	1.00	0.98	0.57	0.88	1.01	0.23	0.20	0.88
**3512**	AT**GCCGTAG**GCGCTAGATGTGC**CGATC**ATAATGCA	0.50	1.00	1.07	0.91	1.10	1.70	0.28	0.27	1.16
**3213**	**GCCGTAG**GTGCGGGAACACTTCAC**CGATC**GATGTT	2.29	1.00	0.93	0.78	1.18	1.22	0.12	0.08	0.94
**510**	**GCCGTAG**TTTCTCGATTCCCAGAGAGA**CGATC**GAT	4.28	1.00	0.98	0.94	1.16	1.36	0.14	0.11	1.04
**59378**	CTG**GCCGTAG**GACAATAGTC**CGATC**CAGATATAGA	0.54	1.00	0.92	0.88	1.20	1.67	0.18	0.23	1.09
**59117**	CTGG**GCCGTAG**GCGATATTAGC**CGATC**CCAGAAGA	1.43	1.00	1.01	1.05	1.15	1.85	0.11	0.11	0.94

Figure S1. 97 sequences with a binding site in common with paionap-5 were analysed for conserved motifs using the MEME suite. The analysis revealed two conserved motifs, the first (**A**) present in all 97 sequences and the second (**B**) present in 92 sequences.

A

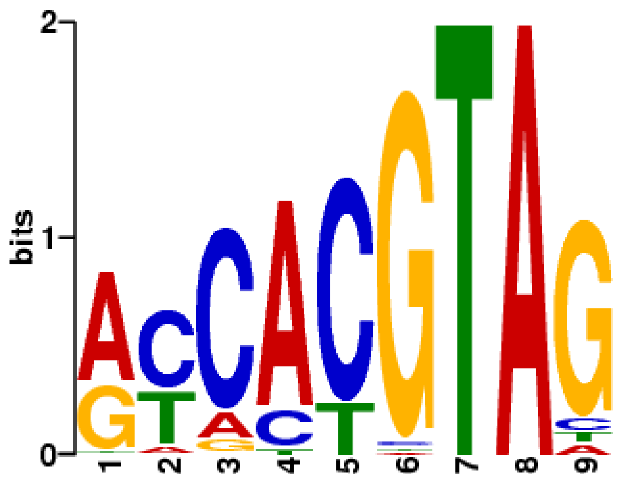

B

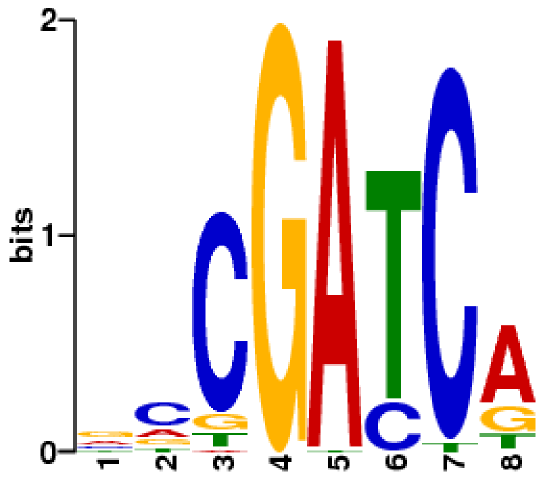

Figure S2. 82 sequences with a binding site in common with paionap-40 were analysed for conserved motifs using the MEME suite. The analysis revealed two conserved motifs shown in (**A**) and (**B**), both present in 74 sequences.

A

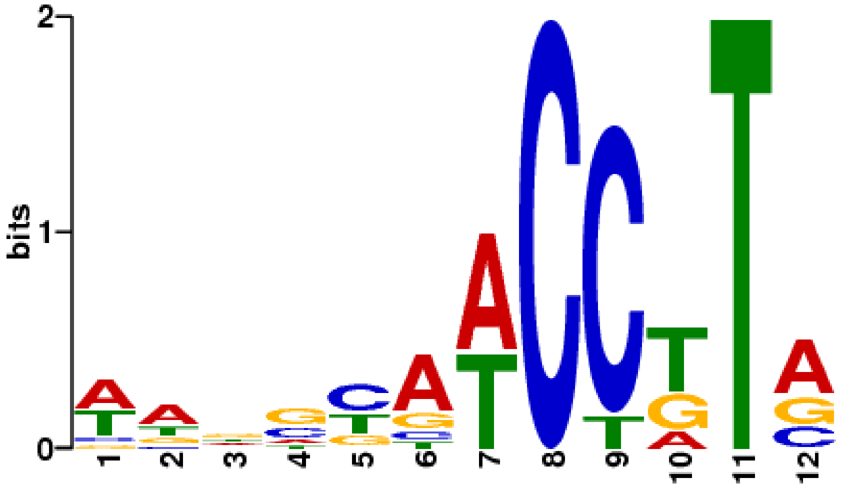

B

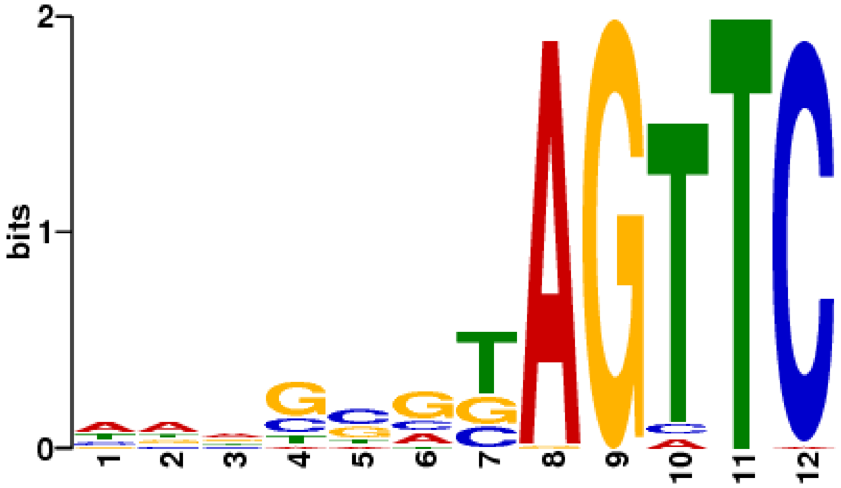

Figure S3. SPR analysis showing the binding profiles of truncated aptamers P5.46 and P40.49 to PAI-1 mutants. To circumvent variation originating from the small differences in active PAI-1 content for different PAI-1 preparations, we compared the concentration-independent dissociation phases after PAI-1:aptamer complex formation. Around 25 RU of biotinylated P5.46 (**A**) and P40.49 (**B**) were captured on SPR sensor surfaces with 500 RU of immobilised streptavidin. PAI-1 wild type (wt) and alanine mutants (K71A, R78A, Y81A, K82A, F116A, R120A, K124A) were subsequently injected (at 5-20 nM) over the sensor surfaces. Shown are the dissociation phases after PAI-1:aptamer complex formation, normalised to the observed PAI-1 binding level with each PAI-1 variant. Dissociation phases for PAI-1 mutants R78A, F116A and R120A have been omitted for clarity and were either comparable to or faster than observed with K82A (P40.49) or could not be determined due to low binding levels (P5.46). The RNA sequences of P5.46 and P40.49 are 5´-GGACAUUGAACCACGUAGGCUCGUUUCUGAGCCGAUCUCGAUGUUCA-3´ and 5´-GGACGACAUUUAUCGAAUUGAUAACCUUACGCGAGAGCGUAGUUCGUUCA-3´, respectively. Minor variations to the 5´- and 3´-ends were made to initiate transcription with a G increasing transcriptional yield. A 3´-A was added to allow ribose oxidation during the biotinylation procedure.

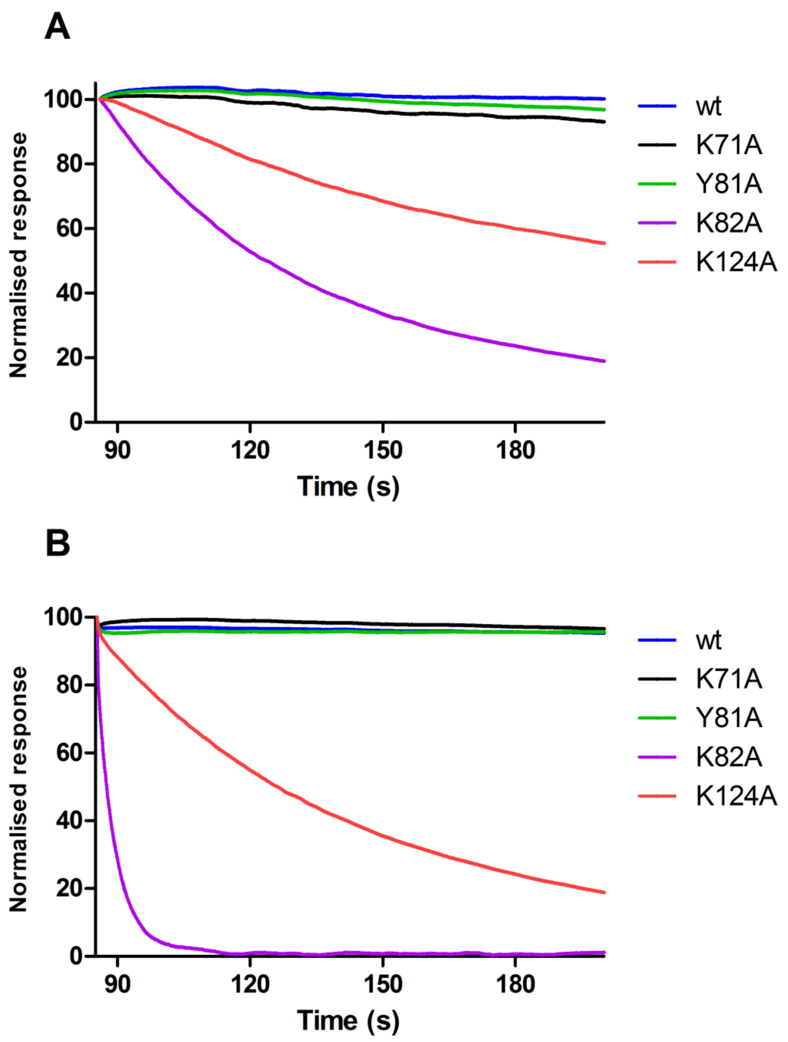

Figure S4. MEME motif search result for Phe116-Arg120 binding sequences. Sequences with a binding pattern encompassing residues Phe116 and Arg120 in PAI-1 but not Lys71, Arg78, Tyr81, Lys82 and Lys124 were analysed for conserved motifs using the MEME suite. The analysis revealed the two conserved motifs shown in (**A**) and (**B**), present in more than 90% of the sequences.

A

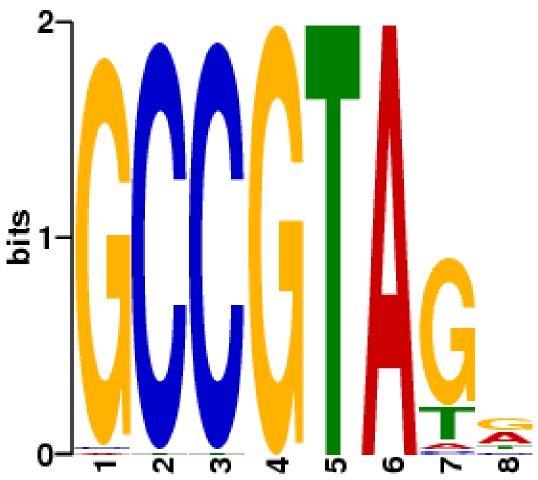

B

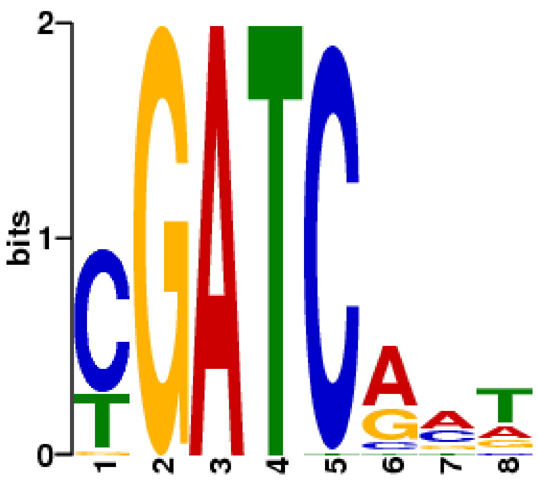

Figure S5. Conserved motifs in the SEQ ID 58088 and 415 groups. Upon screening the input RNA pool for binding to the PAI-1:vitronectin complex the one thousand sequences with highest enrichment factors were subjected to a motif search using the MEME Suite. Among the sequences with highest enrichment factors two conserved motifs were dominating, the SEQ ID 58088 group (**A**) and the SEQ ID 415 group (**B**).

A

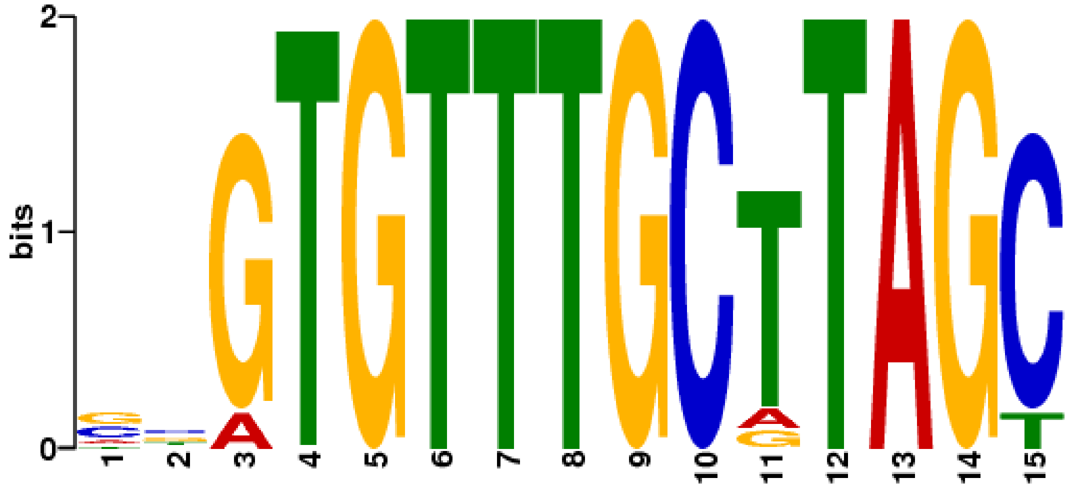

B

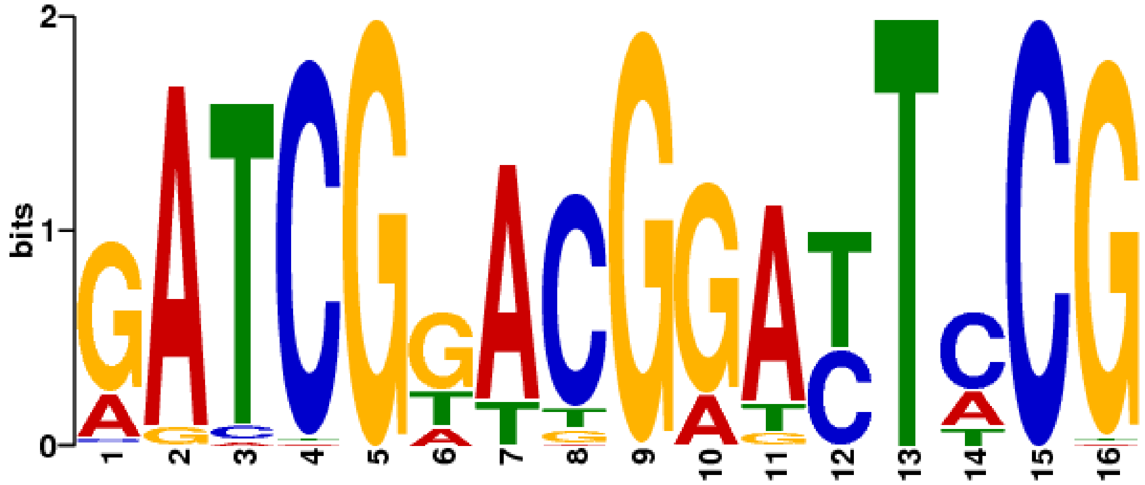

Figure S6, SPR analysis of paionap-5, paionap-40, SEQ ID 415 and SEQ ID 58088 binding to the PAI-1:VN complex. (**A-D**) Biotinylated RNA (20-40 RU) was captured on streptavidin sensor surfaces. Five nM PAI-1 (HT-1080) alone (black curves) or in the presence of 50 nM vitronectin (grey curves) was subsequently passed over the immobilised paionap-5 (**A**), paionap-40 (**B**), SEQ ID 415 (**C**) or SEQ ID 58088 (**D**). Representative sensorgrams are shown for the association and dissociation of injected PAI-1 samples. Vitronectin interferes with the binding of paionap-5 and -40, but not SEQ ID 415 and 58088 to PAI-1. (**E**) The results were confirmed by the inverse setup, where vitronectin was coupled to the sensor surface and PAI-1 captured onto the vitronectin. When passing 100 nM of RNA over the PAI-1:vitronectin surface paionap-5 (red curve) and -40 (blue curve) were unable to bind, whereas SEQ ID 415 (black curve) and 58088 (green curve) bound the complex. A representative sensorgram depicts the RNA association and dissociation phases.

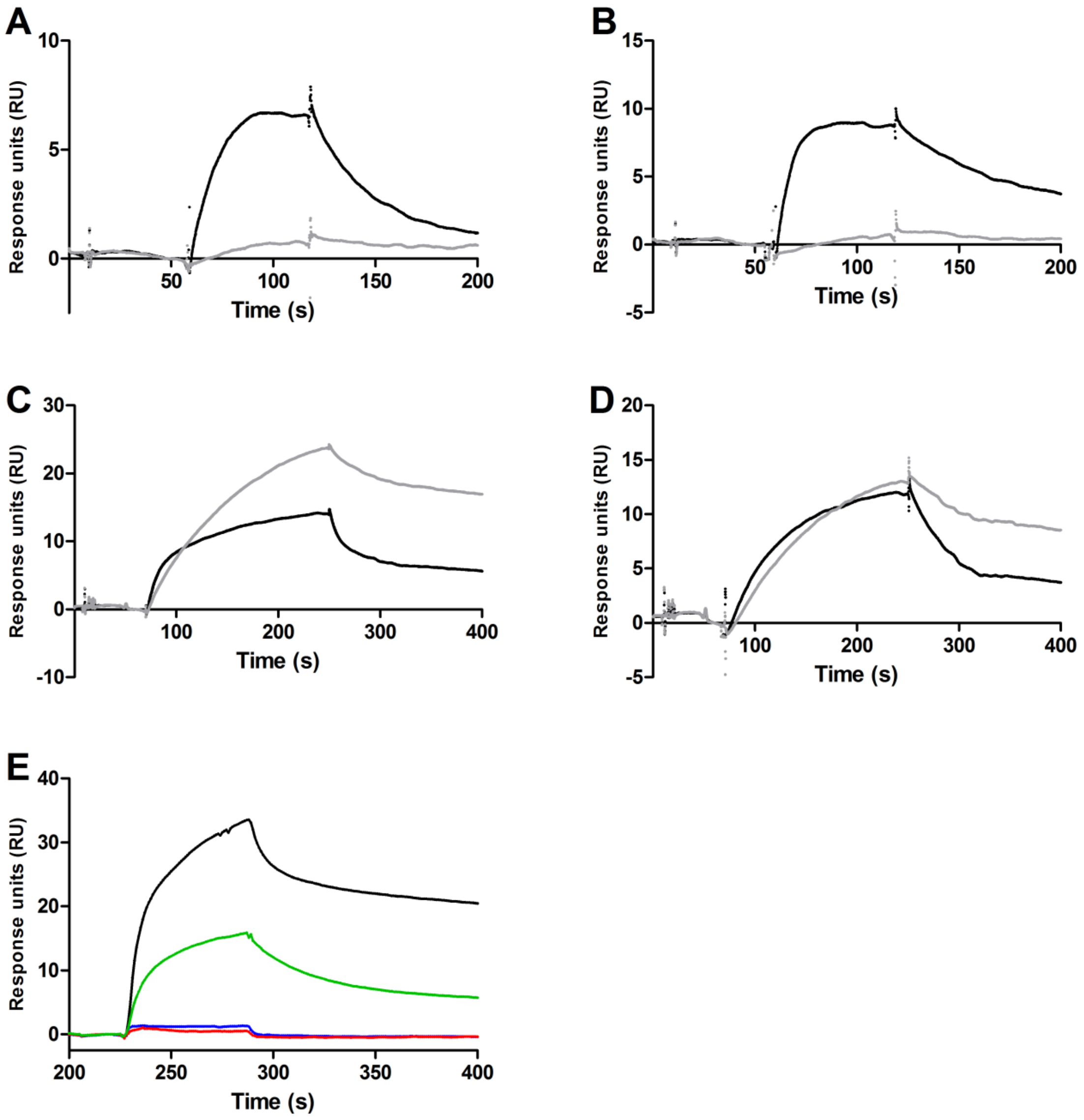

Supplement: SUPPLEMENTARY DATA [file supp_gkv700_nar-00813-met-f-2015-File009.pdf]
